# Germline ablation achieved via CRISPR/Cas9 targeting of *NANOS3* in bovine zygotes

**DOI:** 10.3389/fgeed.2023.1321243

**Published:** 2023-11-27

**Authors:** Maci L. Mueller, Bret R. McNabb, Joseph R. Owen, Sadie L. Hennig, Alba V. Ledesma, Mitchell L. Angove, Alan J. Conley, Pablo J. Ross, Alison L. Van Eenennaam

**Affiliations:** ^1^ Department of Animal Science, University of California, Davis, Davis, CA, United States; ^2^ Department of Population Health and Reproduction, School of Veterinary Medicine, University of California, Davis, Davis, CA, United States

**Keywords:** germline ablation, NANOS3, bovine, embryo, gene editing, CRISPR/Cas9

## Abstract

*NANOS3* is expressed in migrating primordial germ cells (PGCs) to protect them from apoptosis, and it is known to be a critical factor for germline development of both sexes in several organisms. However, to date, live *NANOS3* knockout (KO) cattle have not been reported, and the specific role of *NANOS3* in male cattle, or bulls, remains unexplored. This study generated *NANOS3* KO cattle *via* cytoplasmic microinjection of the CRISPR/Cas9 system *in vitro* produced bovine zygotes and evaluated the effect of *NANOS3* elimination on bovine germline development, from fetal development through reproductive age. The co-injection of two selected guide RNA (gRNA)/Cas9 ribonucleoprotein complexes (i.e., dual gRNA approach) at 6 h post fertilization achieved a high *NANOS3* KO rate in developing embryos. Subsequent embryo transfers resulted in a 31% (*n* = 8/26) pregnancy rate. A 75% (*n* = 6/8) total KO rate (i.e., 100% of alleles present contained complete loss-of-function mutations) was achieved with the dual gRNA editing approach. In *NANOS3* KO fetal testes, PGCs were found to be completely eliminated by 41-day of fetal age. Importantly, despite the absence of germ cells, seminiferous tubule development was not impaired in *NANOS3* KO bovine testes during fetal, perinatal, and adult stages. Moreover, a live, *NANOS3* KO, germline-ablated bull was produced and at sexual maturity he exhibited normal libido, an anatomically normal reproductive tract, and intact somatic gonadal development and structure. Additionally, a live, *NANOS3* KO, germline-ablated heifer was produced. However, it was evident that the absence of germ cells in *NANOS3* KO cattle compromised the normalcy of ovarian development to a greater extent than it did testes development. The meat composition of *NANOS3* KO cattle was unremarkable. Overall, this study demonstrated that the absence of *NANOS3* in cattle leads to the specific deficiency of both male and female germ cells, suggesting the potential of *NANOS3* KO cattle to act as hosts for donor-derived exogenous germ cell production in both sexes. These findings contribute to the understanding of *NANOS3* function in cattle and have valuable implications for the development of novel breeding technologies using germline complementation in *NANOS3* KO germline-ablated hosts.

## 1 Introduction

The transmission of genetic information across generations relies on the integrity of the germline. During fetal development, primordial germ cells (PGCs) are specified as the embryonic precursors of mature germ cells, which ultimately give rise to spermatozoa or oocytes. PGCs are initially established in extraembryonic regions and must undergo migration to the developing gonads, where they will enter a period of mitotic proliferation, undergo meiosis and eventually differentiate into fully mature gametes. Gametes carry the genetic material necessary for the formation of a new individual upon their fusion after mating. As the embryo develops, new PGCs are formed, thus perpetuating the germline cycle (Dechiara et al., 2009).

The *NANOS* gene family plays a crucial role in germ cell development across diverse organisms. Initially discovered in *Drosophila* embryos, the *nanos* gene encodes a protein necessary for the development of both male and female germlines (Wang and Lehmann, 1991). In mammals, three homologs of *NANOS* genes have been identified, two of which exhibit specific expression in germ cells (Tsuda et al., 2003). *NANOS2* is predominantly expressed in male germ cells and is essential for maintaining the spermatogonial stem cell (SSC) population. However, it is not required for female germline development or fertility (Tsuda et al., 2003). Recently, *NANOS2* knockout (KO) pigs, sheep, and cattle were found to copy the phenotype of *NANOS2* KO mice with male specific germline ablation and normal female germline development (Park et al., 2017; Ciccarelli et al., 2020; Latham et al., 2023).

Compared to *NANOS2*, *NANOS3* exhibits earlier expression in developing embryos, as it is predominantly found in migrating PGCs, where it plays a critical role in protecting them from apoptosis. The loss of *Nanos3* in mice leads to the complete absence of both male and female germ cells (Tsuda et al., 2003; Suzuki et al., 2007). Furthermore, decreased levels of *NANOS3* in human cells have been associated with a reduction in both germ cell numbers and expression of genes involved in germ cell regulation (Julaton and Reijo Pera, 2011). Additionally, studies involving *NANOS3* KO livestock have further demonstrated the conserved role of *NANOS3* in germline development. Male and female *NANOS3* KO pigs exhibited a complete loss of germ cells, while their gonadal development remained normal (Kogasaka et al., 2022; Park et al., 2023; Wang et al., 2023). Similarly, in cattle, researchers produced a *NANOS3* KO female fetus through somatic cell nuclear transfer (SCNT) cloning, which also showed a complete absence of germ cells but normal gonadal development (Ideta et al., 2016). However, in this study no live cattle were produced, and the impact of *NANOS3* elimination on male bovine germline development was not reported (Ideta et al., 2016).

The objectives of this study were to optimize a gene KO approach of *NANOS3* using the CRISPR/Cas9 system in bovine zygotes*,* generate pregnancies using *NANOS3* KO embryos, and evaluate the effect of disrupting *NANOS3* on bovine germline development from fetal development through reproductive age. By investigating the consequences of *NANOS3* disruption, this study aimed to advance the understanding of bovine germline development and inform the development of improved livestock breeding strategies.

## 2 Material and methods

### 2.1 Animal care

All experiments using animals were performed in accordance with the University of California (UC), Davis, Institutional Animal Care and Use Committee (IACUC) approved protocol #21513. Cattle were housed and managed at the UC Davis Beef Barn and Feedlot.

### 2.2 *NANOS3* guide RNA (gRNA) design and *in vitro* testing

Single guide RNAs (sgRNA) targeting bovine *NANOS3* exon one were designed using sgRNA Scorer 2.0 (Chari et al., 2017) and Cas-OFFinder (Bae et al., 2014). Based on a systematic analysis of CRISPR/Cas9 mismatch tolerance (Anderson et al., 2015) and previous experiments in bovine zygotes (Hennig et al., 2020), only sgRNAs that met specific mismatch parameters were selected for testing. A mismatch was defined as a discrepancy between a base of the sgRNA and a predicted off-target site. sgRNA selection was undertaken with two requirements; 1) at least three total mismatches between the sgRNA and predicted off-target sequences and 2) at least one mismatch in the seed region (8–11 bp upstream of the protospacer adjacent motif (PAM) site). sgRNAs (Supplementary Table S1) were ordered from Synthego (Menlo Park, CA) and tested *via* *in vitro* cleavage assays comprising 80 ng of polymerase chain reaction (PCR) amplified target sequence, 100 ng of sgRNA, and 150 ng of Cas9 protein (PNA Bio, Newbury Park, CA), in 1× Buffer 3.1 (New England Biolabs, Ipswich, MA), incubated at 37°C for 1 h. The resulting cleavage products were electrophoresed and imaged using a 2% agarose gel.

### 2.3 Bovine embryo production

Bovine ovaries were obtained from an abattoir and transported to the laboratory in 35°C–37°C sterile saline. Collection of cumulus-oocyte complexes (COCs) was performed *via* aspiration of follicles. Groups of 50 COCs were matured in four-well dishes containing 500 μL of maturation media (BO-IVM, IVF Bioscience, Falmouth, United Kingdom). COC maturation was performed in a humidified 5% CO_2_ incubator at 38.5°C for 18–22 h. Oocytes in groups of 25 per drop (60 μL) of SOF-IVF (Bakhtari and Ross, 2014) covered with OVOIL (Vitrolife, Sweden) were fertilized using conventional or Y-sorted, frozen semen (see Table 2). A concentration of 2 × 10^6^ sperm per mL was used for an incubation period of 6 h at 38.5°C in a humidified 5% CO_2_ incubator. Six hours post fertilization (hpf), presumptive zygotes were denuded of cumulus cells by light vortexing in SOF-HEPES medium (Bakhtari and Ross, 2014) for 5 min. Up to 100 zygotes per well were incubated in 400 μL of culture media (BO-IVC, IVF Bioscience) covered with 300 μL of OVOIL (Vitrolife) at 38.5°C in a humidified atmosphere of 5% CO_2_, 5% O_2_ and 90% N_2_ for 7–8 days.

### 2.4 *NANOS3* gRNA testing - *In vivo*

To determine sgRNA mutation rates, laser-assisted cytoplasmic microinjection (Bogliotti et al., 2016) of presumptive zygotes was performed using 6 pL/zygote of ribonucleoprotein (RNP) complexes, formed by incubating 67 ng/μL of a sgRNA (Synthego) with 167 ng/μL of Cas9 protein (PNA Bio) in Tris-low (0.1 mM) ethylenediaminetetraacetic acid (EDTA) at room temperature for 30 min. Embryos were cultured for 7–8 days and those that reached the blastocyst stage were individually collected and lysed in 10 μL of Epicenter DNA extraction buffer (Lucigen, Palo Alto, CA) at 65°C for 6 min and then 98°C for 2 min.

The target region was amplified by two rounds of nested PCR. The first PCR contained 10 μL GoTaq^®^ Green Master Mix (Promega, San Luis Obispo, CA), 0.4 μL of each primer at 10 μM (*NANOS3*_F1 and *NANOS3*_R1; Supplementary Table S2), and 9.2 μL of DNA in lysis buffer for 3 min at 95°C, 35 cycles of 30 s at 95°C, 30 s at 62°C, and 1 min 72°C, followed by 5 min at 72°C. The second nested PCR was run on 1 μL of the first PCR reaction with 10 μL of GoTaq^®^ Green Master Mix (Promega), 8.2 μL of water, and 0.4 μL of each primer at 10 μM (*NANOS3*_F2 and *NANOS3*_R2; Supplementary Table S2) for 3 min at 95°C, 35 cycles of 30 s each at 95°C, 60°C, and 72°C, followed by 5 min at 72°C. Products were electrophoresed and visualized on 1% agarose gels, and then excised and purified using the QIAquick Gel Extraction Kit (Qiagen, Valencia, CA). Purified PCR products were Sanger sequenced (GENEWIZ, San Francisco, CA), and alignments to the target region were visualized with SnapGene (Dotmatics, San Diego, CA) and further analyzed using Synthego’s Inference of CRISPR Edits (ICE) tool (Conant et al., 2022).

Mutation rates for dual gRNA combinations were determined using the same methods as described above except two sgRNAs (67 ng/μL each; Synthego) were incubated together with Cas9 protein (PNA Bio) at room temperature in Tris-low (0.1 mM) EDTA buffer for 30 min prior to microinjection using 6 pL of the RNP mixture.

### 2.5 Embryo transfers (ET)

Estrus synchronization of recipient cattle was initiated 16 days before the scheduled ET date. On day 0, recipients received an intravaginal progesterone (1.38 g) releasing device (EAZI-BREED™ CIDR^®^ (controlled internal drug release); Zoetis, Parsippany, NJ) and gonadorelin (100 μg; Factrel; Zoetis). On day 7, CIDRs were removed and prostaglandin (25 mg; Lutalyse; Zoetis) was administered. Recipients were monitored for signs of estrus using heat patches and visual observation. A second dose of gonadorelin (100 μg; Factrel; Zoetis) was given on day 9. Also, on day 9 of synchronization, presumptive zygotes were microinjected with dual gRNA_4 + 7 (sgRNA_4 and sgRNA_7 combined) RNP complexes as described above. Embryos were microinjected in groups of 50–60, and fresh RNP complexes were prepared between each group. Recipient synchronization was confirmed on day 15 *via* detection of a corpus luteum using a transrectal ultrasound (5.0 MHz linear probe; EVO Ibex, E.I. Medical Imaging, Loveland, CO). ETs were performed on day 16 of recipient synchronization. A caudal epidural of 100 mg 2% lidocaine (Xylocaine; Fresenius, Germany) was administered to recipients prior to ET. Straws (0.25 cc) were loaded with one to two blastocysts each and transferred into the uterine horn ipsilateral to the corpus luteum using a non-surgical transcervical technique. Any remaining blastocysts that were not transferred were analyzed *via* PCR and Sanger sequencing as described previously to get an editing profile of contemporary embryos produced on the same day as those that were transferred to recipients. On day 28 of embryonic development, blood was drawn from the recipients to diagnose pregnancy via pregnancy-associated glycoprotein detection. Transrectal ultrasonography was used to confirm pregnancies on day 35 of embryonic development, to determine fetal sex between 50 and 70 days of embryonic development, and periodically thereafter to monitor pregnancies until delivery.

### 2.6 Sample collection

Bovine gonadal samples resulting from transferred blastocysts that were presumptively edited were collected at four different stages of development: 41-day-of-fetal-age (41 days fetuses), 90-day-of-fetal-age (90 days fetuses), 283-day-of-fetal-age (283 days perinate or birth), and 15-month-of-age (15 months cattle). To collect 41 days and 90 days fetuses, recipient cattle were slaughtered via penetrating captive bolt and subsequent exsanguination. The reproductive tracts were collected, and fetuses were recovered from the uterine horns. Fetuses were phenotyped for crown rump length and sex, and tail tissue samples were collected for DNA extraction. Fetal gonadal ridges (41 days) and gonads (90 days) were identified based on their location within the abdominal cavity, anatomy, and relationship with neighboring organs (mesonephros and/or kidneys) and were collected and preserved for analysis (described below). Age-matched control wildtype (WT) fetal samples, produced via artificial insemination, were also collected in the same manner. In this study, WT refers to the genetically WT form of *NANOS3* (i.e., *NANOS3* ^+/+^), which represents the natural, non-mutated state of the gene. For the 283 days perinate sample collection, blood and gonads were collected during the necropsy of a full-term, stillborn calf. Around 15 months of age, live cattle were slaughtered *via* penetrating captive bolt and meat samples and reproductive tracts were collected. Meat samples were analyzed by proximate analysis and for minerals. Reproductive tracts were analyzed for abnormalities and gonads were isolated and preserved for analysis (described below). Age-matched (283 days perinate and 15 months cattle) control WT gonads were collected as part of separate ongoing departmental experiments. DNA extraction was performed using Qiagen’s DNeasy Blood and Tissue Kit according to the manufacturer’s protocol for tissue (41 days and 90 days fetuses) and blood (283 days perinate and live calves).

### 2.7 Genotypic analysis of bovine samples

#### 2.7.1 Fetal sex genotype determination

The sex chromosome makeup of fetuses were assayed by PCR of the DEAD box helicase 3 gene (DDX3X/DDX3Y), as described by Gokulakrishnan et al. (2012), using 200 nM of each primer (DDX3_F and DDX3_R; Supplementary Table S2) in GoTaq^®^ Green Master Mix (Promega). Reactions were incubated for 3 min at 95°C, 35 cycles of 30 s each at 95°C, 55°C, and 72°C, followed by 5 min at 72°C. Products were electrophoresed and visualized on 2% agarose gels. Amplicon size allowed discrimination between X and Y chromosomes (208 bp *versus* 184 bp, respectively). Genomic DNA from adult testes and ovaries were used as male and female controls.

#### 2.7.2 Short-range PCR of bovine *NANOS3*

The *NANOS3* exon one target region was amplified by PCR of 100 ng of DNA using 200 nM of each primer (*NANOS3*_F2 and *NANOS3*_R2; Supplementary Table S2) in GoTaq^®^ Green Master Mix (Promega) incubated at 95°C for 3 min, 35 cycles of 30 s each at 95°C, 60°C, and 72°C, followed by 5 min at 72°C. Products were electrophoresed and visualized on 1% agarose gels, and then excised and purified using the QIAquick Gel Extraction Kit (Qiagen). Purified PCR products were Sanger sequenced (GENEWIZ), and alignments to the target region were visualized with SnapGene (Dotmatics) and further analyzed using ICE (Synthego) (Conant et al., 2022).

#### 2.7.3 Long-range PCR of bovine *NANOS3*

A 6,274 bp region centered around the *NANOS3* dual gRNA_4 + 7 target location was amplified by long-range PCR using 12.5 μL of Phusion^®^ High-Fidelity PCR Master Mix (Thermo Fisher Scientific Inc., Waltham, MA), 50 ng of DNA, 10 μL of water, 200 nM of each primer (*NANOS3*_6 kb_2F and *NANOS3*_6 kb_2R; Supplementary Table S2), and 0.5 μL of dimethyl sulfoxide (DMSO) for 3 min at 98°C, 35 cycles of 15 s at 98°C, 30 s at 65°C, and 6 min 72°C, followed by 6 min at 72°C. Long-range PCR products were visualized on a 1% agarose gel.

#### 2.7.4 *NANOS3* long-amplicon library preparation, sequencing, and evaluation

Long-range PCR products were purified using an AMPure PB Kit (Pacific Biosciences of California (“PacBio”), Menlo Park, CA) following the manufacturer’s protocol. SMRTbell libraries were prepared with PacBio barcoded overhang adapters, which allowed for pooling of the samples (SMRTbell^®^ Express Template Prep Kit 2.0 and Barcoded overhang adapter kit 8A, PacBio). Sequencing was performed on a PacBio Sequel II system by the UC Davis DNA Technologies & Expression Analysis Core. HiFi reads (reads generated with Circular Consensus Sequencing (CCS) analysis whose quality value is equal to or greater than 20) were sorted by barcode and BAM files were converted to individual FASTQ files for each sample using SMRT Link v11.0.0.146107. HiFi reads were aligned to a reference FASTA file corresponding to the 6,274 bp target region of bovine *NANOS3* (ARS-UCD1.2-Ensembl version 108: Chr7:11,805,072–11,811,345) using the “MEM” algorithm implemented in the BWA MEM2 v2.2.1 software (Vasimuddin et al., 2019). SAM files were converted to BAM files and sorted and indexed using SAMtools v1.15 (Danecek et al., 2021). The resulting BAM files were used as input for the variant determination algorithm (.batch) implemented in AlleleProfileR (Bruyneel et al., 2019) to define and count alleles present in each sample.

### 2.8 Phenotypic analysis of bovine samples

At 41 days (*n* = 2 *NANOS3*-presumptively-edited and 1 WT control), the whole urogenital ridge was isolated and preserved for histology. Whole fetal testes were isolated from the 90 days fetuses (*n* = 2 *NANOS3*-presumptively-edited and 2 WT control). One testis from each fetus was preserved for histology while the other testis was preserved for single-cell RNA-sequencing (scRNA-Seq) analysis. Testicular cross-sections from the 283 days perinates (*n* = 1 *NANOS3*-presumptively-edited and 1 WT control) were collected and preserved for both histology and scRNA-Seq analysis. Testicular cross-sections from the 15 months bulls (*n* = 2 *NANOS3*-presumptively-edited and 1 WT control) and heifer (n = 1 *NANOS3*-presumptively-edited) were also collected and preserved for histology as described below.

#### 2.8.1 scRNA-Seq

##### 2.8.1.1 Gonad preservation and dissociation for scRNA-Seq

Single cells were isolated from whole 90 days fetal testes and cross-sections of equal weight from 283 days perinatal testes. Gonadal samples collected for scRNA-Seq were washed in ice-cold phosphate-buffered saline (PBS) and slow frozen in Dulbecco’s Modified Eagle’s Medium (DMEM) containing 20% fetal bovine serum (FBS) and 10% DMSO using a freezing device (Mr. Frosty™, Thermo Fisher Scientific) (Soto and Ross, 2021). Cryovials of slow-frozen gonadal samples were removed from liquid nitrogen storage and thawed at 37°C in a water bath until the tissue could be removed (maximum of 3 min). Thawed gonads were rinsed in room temperature Hank’s Balanced Salt Solution with calcium and magnesium (HBSS +/+) and minced into ∼0.1 mm pieces.

Perinatal samples were subject to a two-step digestion procedure, as described by Guo et al. (2017) and Guo et al. (2018), with modifications optimized for bovine samples. Gonads were first digested in a pre-warmed mixture of HBSS +/+ containing collagenase type IV (1 mg/ml) Sigma-Aldrich Roche^®^, Burlington, MA) and DNase I (1 kU/mL) (Sigma-Aldrich Roche^®^) for 5 min at 37°C with gentle agitation (250 rpm), then shaken vigorously for 1 min and incubated for another 3–5 min with gentle agitation. The dissociated tubules were sedimented by centrifugation at 600 *g* for 5 min at 4°C and washed with HBSS without calcium or magnesium (HBSS −/−). The pellet was resuspended in a second pre-warmed digestion media of 5 ml of 0.25% trypsin/EDTA supplemented with DNase I (1 kU/mL). The suspension was pipetted vigorously three to five times with a wide-bore pipette and incubated at 37°C for 5 min. The process was repeated in 5 min increments for up to 15 min total. The digestion was stopped by adding 10% FBS. Single testicular cells were obtained by filtering through strainers with mesh size 100 μm and 30 µm. The cells were pelleted by centrifugation at 600 *g* for 15 min at 4°C and washed with ice-cold HBSS −/−. Cells were then re-suspended in ice-cold HBSS −/− supplemented with 0.4% Bovine Serum Albumin (BSA).

Fetal samples were digested in a pre-warmed mixture of HBSS +/+ containing collagenase type IV (1 mg/ml) and DNase I (5 kU/mL). The suspension was triturated vigorously three to five times with a wide-bore pipette and incubated at 37°C for 5 min with gentle agitation (150 rpm). The process was repeated three to five times in 5 min increments for up to 25 min total. The digestion was stopped by adding 10% FBS. Single cells were obtained by filtering through strainers with mesh size 100 μm and 30 µm. The cells were pelleted by centrifugation at 600 *g* for 15 min at 4°C. Cells were then re-suspended in ice-cold HBSS −/− supplemented with 0.4% BSA.

##### 2.8.1.2 scRNA-Seq library preparation, sequencing, and analysis

Single cell samples were processed using the cell fixation (SB1001) and single-cell whole-transcriptome (SB 2001) kits from Parse Biosciences (Seattle, WA), according to the manufacturer’s instructions. This scRNA-Seq approach is based on combinatorial barcoding, which enables multiplexing of samples. The resulting sub-libraries (*n* = 8) were sequenced on an Illumina NovaSeq 6000 instrument (150 base paired end). For data processing, the Parse Bioscience’s processing pipeline (v0.9.6p) was used with default settings to demultiplex samples and align reads to the bovine reference genome (ARS-UCD1.2-Ensembl version 105). Downstream analysis was performed using the R package Seurat (v4.1.0) at default settings unless otherwise noted (Stuart et al., 2019).

Individual analyses were performed for each sample timepoint. For the 90 days fetal testes (*n* = 4), to be included in the analysis, a cell had to have between 1,000 and 100,000 reads from at least 700 genes, and the genes had to be expressed in more than 10 cells. To be included in the analysis for the 283 days perinatal testes (*n* = 2), a cell had to have between 500 and 50,000 reads in at least 200 genes, and similarly the genes had to be expressed in more than 10 cells. For both timepoints, the resulting gene–cell matrices were normalized and scaled using Seurat’s NormalizeData and ScaleData functions, and principal component analysis was performed with Seurat’s RunPCA function. Cells were clustered using the FindNeighbors and FindClusters functions. For visualizing clusters, dimensionality reduction was performed by uniform manifold approximation and projection (UMAP). The identities of cell clusters were determined by plotting (VlnPlot & FeaturePlot functions) well-known mammalian germ cell and testicular somatic support cell markers. Once clusters were identified, the FindConservedMarkers and FindMarkers functions (Wilcoxon rank-sum test, minimal fraction of 10%, and log-transformed fold-change threshold of 0.25) were both run on each cluster subset to find genes that were conserved and/or differentially expressed, respectively, between treatments (i.e., KO *versus* control) for each cell type.

#### 2.8.2 Histology

Gonadal samples collected for hematoxylin and eosin (H&E) staining were fixed in 4% paraformaldehyde for 24 h at 4°C. Fixed tissues were rinsed in PBS and then dehydrated through a stepwise ethanol gradient of PBS, 30% ethanol, 50% ethanol, and 70% ethanol. Each step was for 24 h at 4°C. Tissues were stored in 70% ethanol at 4°C until being processed in a Tissue-Tek VIP^®^ processor (Sakura Finetek United States, Inc., Torrance, CA). Tissue was then embedded in paraffin, sectioned at 5 μm thickness and stained with H&E.

Gonadal samples collected for immunostaining analyses were fixed in 4% paraformaldehyde for 6–12 h at 4°C. Fixed tissues were rinsed in PBS and then washed through a stepwise sucrose gradient of 15% sucrose for 24 h and then stored in 30% sucrose, all at 4°C, until being embedded in Tissue-Tek optimal cutting temperature (OCT) compound (Sakura Finetek). The resulting cryoblocks were stored at −80°C prior to sectioning. Cryoblocks were sectioned at 10 μm thickness and tissue sections were stored at −20°C until staining. For immunostaining, room temperature cryosections were washed in Tris-buffered saline (TBS) to remove the OCT compound prior to antigen retrieval. Antigen retrieval was performed in a steamer for 5 min in 10 mM citrate-based antigen unmasking solution (pH 6.0, Vector Laboratories, Newark, CA) for cytoplasmic targets or 20 min in 10 mM Tris-based antigen unmasking solution (pH 9.0, Vector Laboratories) for nuclear targets. Additionally, for nuclear targets, antibody permeability was increased by incubation with 0.1% Triton X-100 in TBS for 10 min. Non-specific binding of immunoglobulins was blocked by incubation with 0.3 M *Glycine* and 10% normal donkey serum for 1 h, at room temperature. Tissue sections were then incubated overnight at 4°C with the following primary antibodies: anti-PRDM1 (1:50; 14–5963–80, Invitrogen, Waltham, MA; AB_1907438), anti-OCT4 (1:100; AF1759, Novus Biologicals, Centennial, CO; AB_354975), and anti-DDX4 (1:500; ab13840, Abcam, Fremont, CA; AB_443012). Also, sections of tissues were incubated with a rabbit isotype control antibody (02–6102, Invitrogen) as the primary antibody to serve as negative control sections. All sections were incubated with appropriate secondary antibodies, anti-rabbit IgG - Alexa Fluor™ 488 (1:500; Invitrogen) or anti-goat IgG - Alexa Fluor™ 568 (1:500, Invitrogen), for 1 h at room temperature. Hoechst 33,342 was used for counterstaining to detect nuclei. Slides were mounted using ProLong Gold Antifade (Invitrogen) and imaged using an Echo Revolve microscope (Discover Echo Inc., San Diego, CA). Images were processed using ImageJ (Fiji v2.3.0/1.53t) (Schindelin et al., 2012).

#### 2.8.3 Live animal evaluation and reproductive examinations

Monthly weights were recorded for the live *NANOS3* edited animals (n = 3) and starting at 5 months old scrotal circumference was measured monthly for males (n = 2). Starting around 12 months of age, at which time bulls typically reach reproductive maturity, breeding soundness exams (BSE) were conducted by UC Davis veterinarians. If a bull failed the first BSE, then two more BSEs were performed at least 1 month apart. BSEs followed the standards set forth by the Society for Theriogenology and included a general physical examination, inspection of reproductive organs, and semen collection *via* electroejaculation (Chenoweth et al., 1993; Chenoweth, 2015). Around 14 months of age, UC Davis veterinarians performed a reproductive exam, including rectal palpation and transrectal ultrasound, on the live heifer (*n* = 1).

#### 2.8.4 Hormone analysis

For hormone analyses, blood samples were collected monthly on the live *NANOS3* edited animals (n = 3) and a WT bull (n = 1). Samples were centrifuged to separate serum and plasma and the serum was stored at −80°C before processing by the UC Davis Clinical Endocrinology Laboratory (School of Veterinary Medicine, Department of Population Health and Reproduction). Radioimmunoassays (RIA) were used to measure serum testosterone (Anti-testosterone, C. Munro, UC Davis) and estradiol (Ultra-Sensitive Estradiol RIA kit #DSL-4800, Beckman Coulter, Brea, CA) levels. Enzyme immunoassays (EIA) were used to measure Anti-Müllerian Hormone (AMH) (Bovine AMH assay #AL-114, Ansh Labs, Webster, TX), inhibin-B (Equine/Canine/Rodent Inhibin-B assay #AL-163, Ansh Labs), and progesterone (Anti-Progesterone-R4859, C. Munro, UC Davis) levels.

#### 2.8.5 Meat analysis

Samples of sirloin cap and chuck arm were dissected from the carcasses of the *NANOS3* KO 15 months heifer #854 and bull #838, trimmed of excess fat, and frozen at −80°C. The muscle samples were analyzed by Midwest Laboratories (Omaha, NE) by proximate analysis and for minerals (Fe, Zn, and P), using AOAC International methods (Rockville, MD) and internally established protocols (Midwest Laboratories; MWL FO 014 & MWL FO 022). Reference nutrient data for beef were taken from Trott et al. (2022).

## 3 Results

### 3.1 CRISPR/Cas9 mediated KO of *NANOS3* in bovine embryos

**FIGURE 1:**
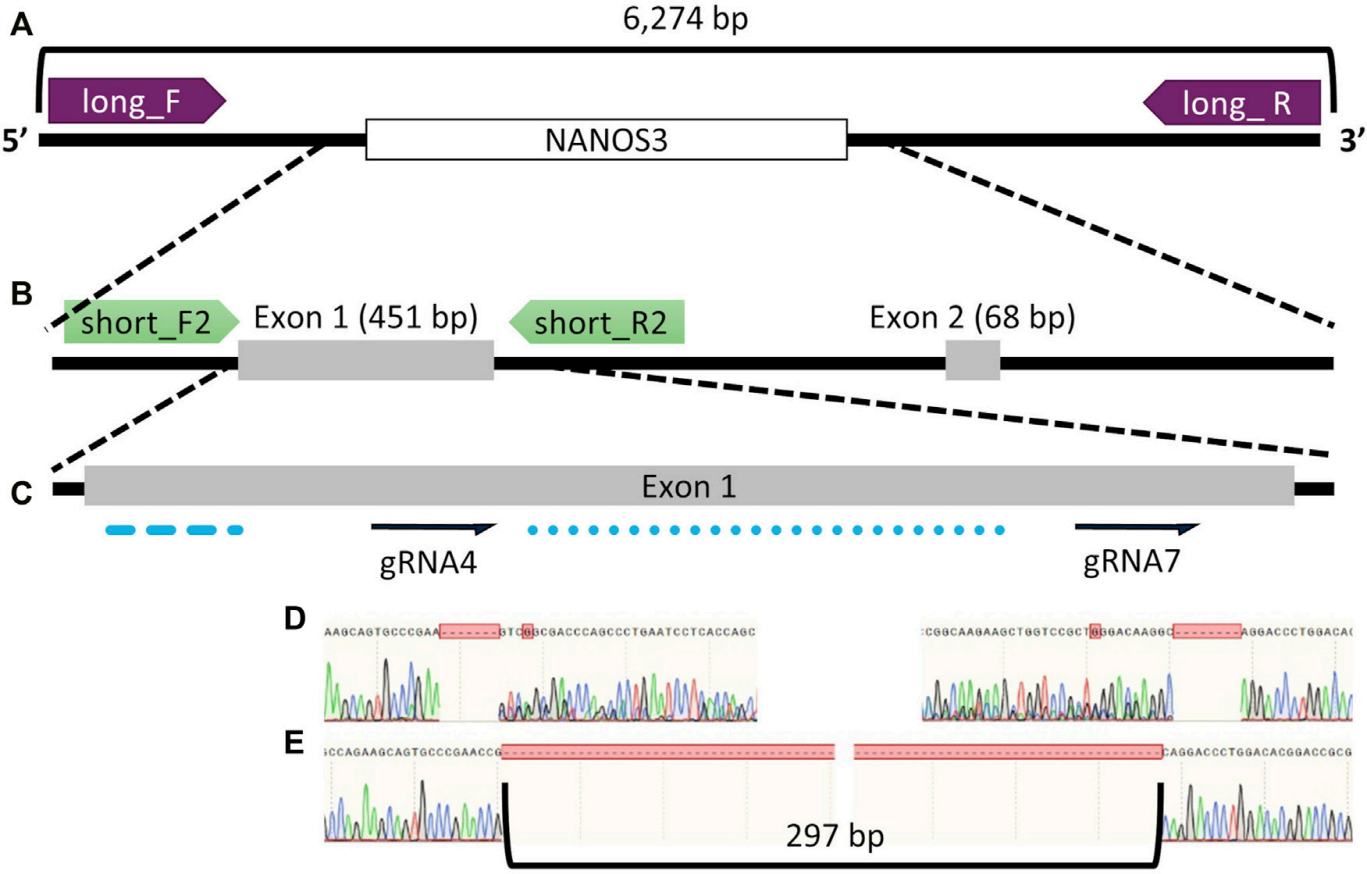
Bovine *NANOS3* targeting and PCR analysis strategy. Diagram of bovine *NANOS3* showing the genomic locations of **(A)** long-range PCR primers (*NANOS3*_6 kb_2F, *NANOS3*_6 kb_2R), **(B)** short-range PCR primers (*NANOS3*_F2, *NANOS3*_R2), and **(C)** selected dual guides, sgRNA4 and sgRNA7 (dual gRNA_4 + 7) in relation to the highly conserved N-terminal (blue dashed line) and zinc finger (blue dotted line) domains. **(D)** Sanger sequencing results showing representative frameshift mutations from sgRNA4 (left) and sgRNA7 (right), and **(E)** Sanger sequencing results showing a targeted dual gRNA_4 + 7 297 bp deletion.

Seven sgRNAs targeting exon one of the bovine *NANOS3* gene (Supplementary Table S1) were selected based on the specific mismatch criteria previously described. Four sgRNAs efficiently cut the target region *in vitro* (sgRNA #1, #4, #5, and #7), and were then tested *in vivo via* embryo microinjection. Each sgRNA was incubated with Cas9 protein to form a RNP complex and independently microinjected into zygotes (n = 30 embryos/sgRNA) 6 hpf, following a previously established protocol (Hennig et al., 2020). Non-injected embryos were cultured to the blastocyst stage as a within experiment, contemporary, developmental control. All microinjected groups had acceptable blastocyst development rates (≥20%), and three sgRNAs (#4, #5, and #7) had an over 60% mutation rate, defined as the end product being different than the starting, WT genome (Table 1). However, none of the sgRNAs achieved over a 75% KO rate. For embryo analysis, a KO was defined as a blastocyst with less than 1% WT alleles detected *via* ICE analysis of Sanger traces. Next a dual gRNA system (i.e., co-injection of two sgRNAs simultaneously) was tested, as this approach has been shown to be an efficient method for complete gene disruption, or KO, in livestock species (Vilarino et al., 2017; Wu et al., 2017). Single guides #4 and #7 (dual gRNA_4 + 7) were selected based on their mutation efficiencies and genomic locations to target both the 5′ and 3’ regions of exon 1 (297 bp between sgRNA cut sites; Figure 1). The dual gRNA_4 + 7 approach achieved an over >75% KO rate and an acceptable blastocyst development rate (19%) (*n* = 22/28, 4 replicates; Table 1).

**TABLE 1:** Comparison of blastocyst development rates and mutation efficiencies of single and dual *NANOS3* gRNAs.

Guide	Location (exon 1)	Replicate	Blastocyst rate	Mutation rate^a^	KO rate^b^
**Control**	-	-	42/120 (35%)	0/10 (0%)	-
**sgRNA1**	5′	-	7/30 (23%)	1/7 (14%)	0/7 (0%)
**sgRNA4**	5′	-	7/30 (23%)	5/6 (83%)	4/6 (67%)
**sgRNA5**	Center	-	6/30 (20%)	5/6 (83%)	2/6 (33%)
**sgRNA7**	3′	-	8/30 (27%)	5/8 (63%)	2/8 (25%)
**Dual sgRNA4 + sgRNA7**	5'& 3′	1	6/30 (20%)	6/6 (100%)	4/6 (67%)
		2	6/30 (20%)	4/6 (67%)	3/6 (50%)
		3	5/40 (13%)	5/5 (100%)	5/5 (100%)
		4	11/50 (22%)	11/11 (100%)	10/11 (91%)
		**Total^c^**	**28/150 (19%)**	**26/28 (93%)**	**22/28 (79%)**

^a^ Mutation: End product being different than the starting, wildtype (WT) genomic sequence.

^b^ Knockout (KO): A blastocyst with less than 1% WT, alleles detected *via* ICE, analysis of Sanger traces.

^c^ Total: Bold values are the cumulative results of all four “Dual sgRNA4 + sgRNA7” replicates.

### 3.2 Generation of *NANOS3* KO cattle

Dual gRNA_4 + 7 *NANOS3* targeted bovine embryos were produced, as described previously, and 26 resulting blastocysts were transferred by collaborating veterinarians into 26 synchronized recipients (Table 2). A 31% pregnancy rate (n = 8/26) was achieved, as confirmed by transrectal ultrasound on day 35 of embryonic development.

**TABLE 2:** Pregnancy results from microinjected bovine embryo transfers (ET).

			Pregnancies				
Rep	ET date	KO rate^a^	28-day	35-day	70-day	Male (#)	Female (#)
**1**	2/27/20	26/26 (100%)	3/8 (38%)	2/8 (25%)	2/8 (25%)	1	1
**2**	5/27/20	n/a^b^	0/6 (0%)	0/6 (0%)	0/6 (0%)	0	0
**3**	6/17/20	n/a^b^	2/5 (40%)	1/5 (20%)	1/5 (20%)	1	0
**4C^c^**	12/16/20	10/12 (83%)	3/4 (75%)	3/4 (75%)	3/4 (75%)	3	0
**4Y^d^**	12/16/20	2/4 (50%)	2/3 (67%)	2/3 (67%)	n/a	2	0
Total^e^		**38/42 (90%)**	**10/26 (38%)**	**8/26 (31%)**	**6/24 (25%)**	**7**	**1**

^a^ Knockout (KO) was defined as a blastocyst with less than 1% WT, alleles detected *via* ICE, analysis of Sanger traces; KO, rate was determined by analyzing the remaining blastocysts that were not used for ET.

^b^ Due to low development, all blastocysts from these replicates were used for ET.

^c^ 4C: Embryos in replicate 4C were *in vitro* fertilized using conventional semen but all resulting pregnancies were male.

^d^ 4Y: Embryos in replicate 4Y were *in vitro* fertilized using male-sex-sorted semen and the resulting fetuses were collected on day 42 of gestation (41 days).

^e^ Total: Bold values are the cumulative result of all five replicates.

**FIGURE 2:**
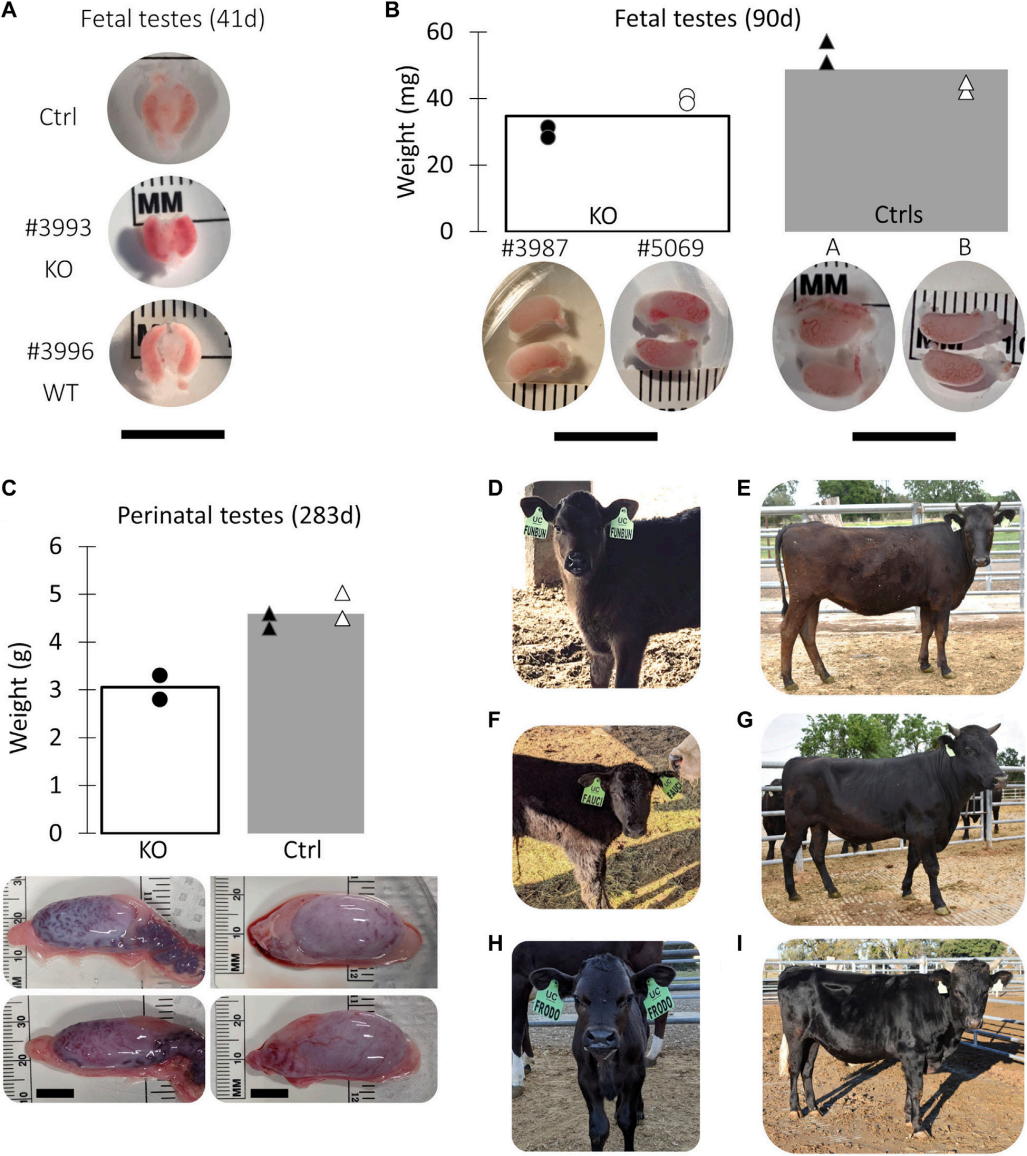
Collection of *NANOS3* targeted bovine samples. **(A)** Images of 41d fetal urogenital ridges. **(B)** Comparison of size and average weight (bars) of testes at 90d of fetal age from *NANOS3* KO (*n* = 4) *versus* control (*n* = 4) fetuses. **(B)** Comparison of size and average weight (bars) of testes at 283d of fetal age from *NANOS3* KO (*n* = 2) *versus* control (*n* = 4) perinates. In both panel **(B, C)**, testes from the same bovine sample (i.e., a testis pair) are indicated by the same shape and color. **(D–I)** Images of live *NANOS3* edited cattle at 1-week-old **(D, F, H)** and 15-months-old **(E, G, I)**. The top row **(D, E)** is heifer #854, middle row **(F, G)** is bull #838, and bottom row **(H, I)** is bull #3964. Scale bars are 1 cm.

A total of 8 pregnancies with *NANOS3* targeted embryos were established. To evaluate *NANOS3* KO bovine fetal gonad development, fetuses were collected at different developmental stages, including during sexual differentiation (41 days; n = 2 *NANOS3*-presumptively-edited and 2 WT) and post sexual differentiation (90 days; n = 2 *NANOS3*-presumptively-edited and 2 WT) (Figures 2A, B). Additionally, one full-term male pregnancy was stillborn, so gonadal samples from the perinatal stage (i.e., during and immediately after birth) were evaluated (283 days; n = 1 *NANOS3*-presumptively-edited and 1 WT) (Figure 2C). Ultimately, three live, healthy calves derived from *NANOS3*-presumptively-edited embryos were born without assistance at the UC Davis Beef Barn, a heifer calf, #854 (“FunBun”) and two bull calves, #838 (“Fauci”) and #3964 (“Frodo”) (Figures 2D–I). These calves were grown and developed for analysis at reproductive age (∼12 months of age) and finally were harvested at 15 months to examine their reproductive tracts. Additionally, meat samples were collected from the *NANOS3* KO cattle, heifer #854 and bull #838, at harvest for compositional analysis.

### 3.3 Genotypic analysis of CRISPR/Cas9 *NANOS3* targeted bovine samples

**FIGURE 3:**
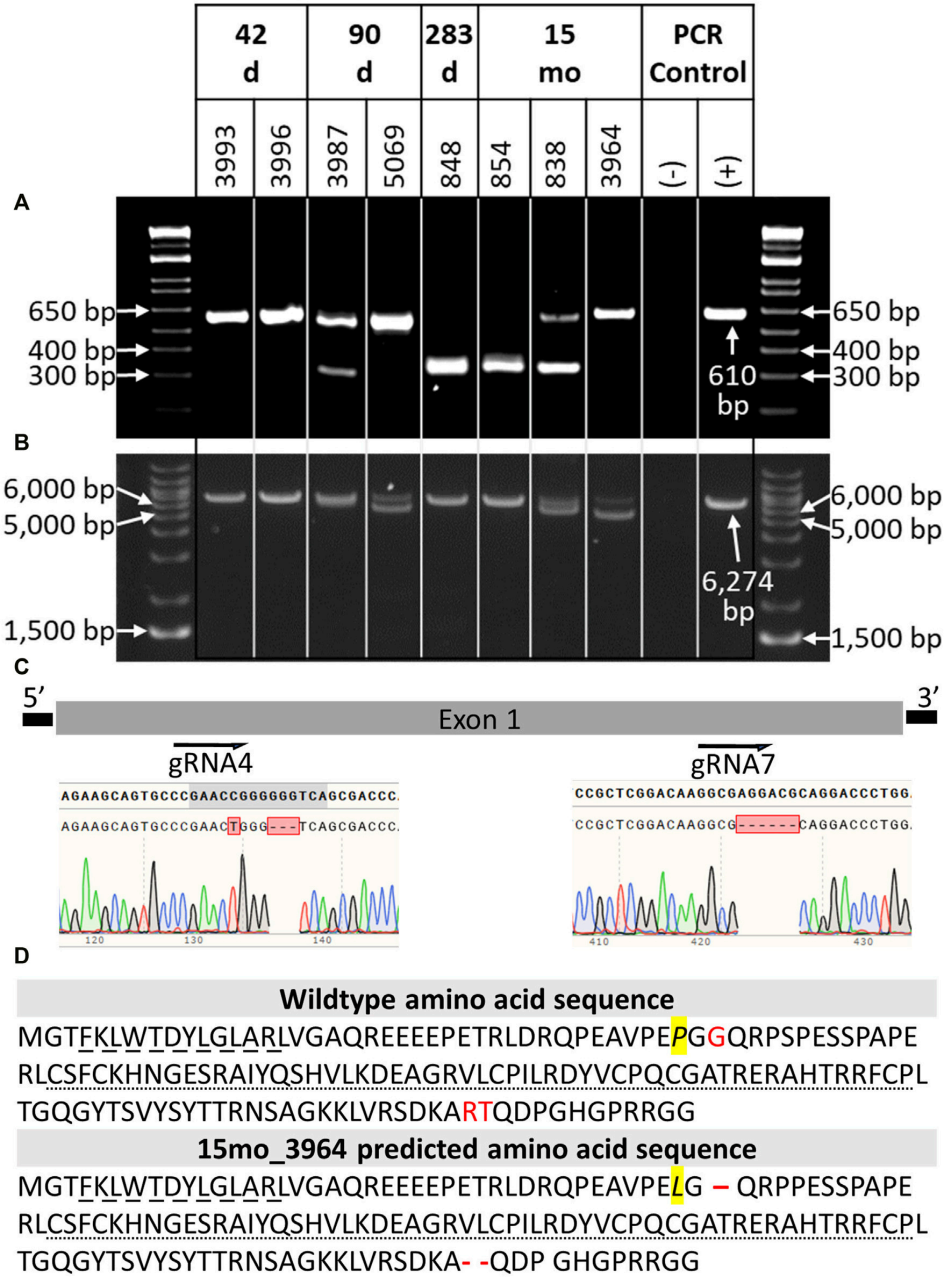
Genotypic analysis of CRISPR/Cas9 *NANOS3*-presumptively-edited bovine samples (*n* = 8). **(A)** *NANOS3* PCR results using short-range primers (*NANOS3*_2F, *NANOS3*_2R). **(B)** *NANOS3* PCR results using long-range primers (*NANOS3*_6 kb_2F, *NANOS3*_6 kb_2R). Genetic wildtype (WT; +) band sizes are 610 bp **(A)** and 6,274 bp **(B)**. **(C)** Diagram showing the Sanger sequencing results of small, in-frame mutations present in one of the edited *NANOS3* alleles carried by 15 months_3964. There was a single bp substitution (C to T) and a 3 bp deletion near the sgRNA4 cut site and a 6 bp deletion near the sgRNA7 cut site. **(D)** Comparison of the WT bovine *NANOS3* exon one amino acid sequence to 15 months_3964’s predicted amino acid sequence. The amino acid substitution is highlighted in yellow and italicized (P to L). The three deleted amino acids are represented by red font (WT) and dashes (15months_3964). The highly conserved N-terminal (dashed underline) and zinc finger binding (dotted underline) domains are underlined.

DNA was extracted from tail tissue (41 days and 90 days fetuses) or blood (283 days perinate and live calves). Seven of the eight samples were determined to be male by PCR of DDX3X/DDX3Y. Initial *NANOS3* genotypes were determined by short-range PCR amplification (Figure 3A) and Sanger sequencing of the *NANOS3* exon one target region. This analysis showed that seven of the eight *NANOS3* targeted bovine samples (87.5%) were successfully edited (0% WT alleles remained). One *NANOS3* targeted bovine sample, 41 days_3996, was not edited (100% WT). Four out of the seven edited *NANOS3* bovine samples (57%) appeared to be mosaic (i.e., carried >2 alleles). However, all of the alleles present in the four mosaic samples were predicted to be KO alleles. In this analysis, a KO allele was defined as having either a frameshift-inducing indel (i.e., small indels that are not multiples of three) or an intermediate sized indel (>21 bp) in a protein-coding region that were predicted to generate a complete loss-of-function mutation. Two samples, 283 days_848 and 15 months_854, appeared to be bi-allelic KO (i.e., ≤2 KO alleles and 0% WT alleles) with a homozygous targeted dual gRNA_4 + 7 deletion KO allele, or two unique KO alleles each with targeted dual gRNA_4 + 7 indels (i.e., compound heterozygote), respectively. Finally, one *NANOS3* edited bovine sample, 15 months_3964, appeared to carry only one allele with small, in-frame deletions, and thus was determined to not be a KO allele (Figure 3C). This allele resulted in one amino acid substitution and a deletion of three total amino acids at the target sites (Figure 3D). These mutations were all outside of the highly conserved coding regions for the N-terminal and zinc finger binding domains, and it was unknown whether the deleted amino acids were necessary for *NANOS3* protein function. The exact amino acid sequence that was predicted to result from the mutated allele was not found in any other species when a protein BLAST (Basic Local Alignment Search Tool) analysis was conducted. Overall, six of the seven *NANOS3* edited bovine samples (85.7%) were observed to be successful *NANOS3* KOs, and four (67%) had at least one allele with a targeted dual gRNA_4 + 7 indel.

In order to identify and measure the proportion of alleles present in the mosaic samples and confirm other genotypes, further genotype analysis was completed using long-range PCR amplification and next-generation sequencing on all eight *NANOS3* targeted bovine samples. The long-range PCR was designed to amplify a 6,274 bp region centered around the *NANOS3* dgRNA_4 + 7 target location, and it enabled detection of large (>500 bp) indels. Three of the samples, 90 days_5069, 15 months_838, and 15 months_3964, were observed to carry large (>500 bp) deletions, as indicated by the presence of bands smaller than the WT control sample (6,274 bp; Figure 3B).

The long-range PCR products were submitted for PacBio long-read sequencing, and this data revealed a variety of alleles present in the *NANOS3* targeted bovine samples, with indels ranging from 1 bp up to 1,500 bp (Table 3). Seven of the eight *NANOS3* targeted bovine samples (87.5%) were confirmed to be successfully edited (i.e., 0% WT alleles remained). Additionally, six of the seven *NANOS3* edited bovine samples (85.7%) were confirmed to only carry KO allele(s). Bovine sample 41 days_3993 was found to be a bi-allelic, homozygous KO, carrying only one KO allele with small indels at both sgRNA4 and sgRNA7 cut sites. Bovine sample 283 days_848 was confirmed to be a bi-allelic KO, but two unique KO alleles were found (i.e., compound heterozygote). One of the KO alleles had the targeted dual gRNA_4 + 7 deletion (36% of reads) and the other had an intermediate-sized deletion (−273 bp) near the sgRNA4 cut site and a small deletion (−8 bp) near the sgRNA7 cut site.

**TABLE 3:** Proportion and types of alleles present in the CRISPR/Cas9 *NANOS3*-presumptively-edited bovine samples. Alleles are ordered by the proportion of reads (largest to smallest).

			sgRNA4^a^		sgRNA7^b^		Dual (d) gRNA_4 + 7		
Sample	Allele #	Allele description (indel size category)^c^	Indel start^d^	Indel size (bp)	Indel start^d^	Indel size (bp)	Indel start^d^	Indel size (bp)	Proportion of reads (%)
41d_3993	1	sgRNA 4 & 7: small	118	−1	409	−8			100
41d_3996	1	wildtype							100
90d_3987	1	sgRNA4: intermediate & sgRNA7: small	83	−51	409	−8			37
	2	sgRNA 4 & 7: intermediate	115	−27	410	−32			36
	3	dgRNA_4 + 7: targeted					112	−298	28
90d_5069	1	dgRNA_4 + 7: large					−331	−960	36
	2	dgRNA_4 + 7: large					−662	−1,122	32
	3	sgRNA4: small & sgRNA7: intermediate	107	−20	393	−27			26
	4	sgRNA 4 & 7: intermediate	115	−27	367	−55			5
280d_848	1	sgRNA4: intermediate & sgRNA7: small	116	−273	409	−8			64
	2	dgRNA_4 + 7: targeted					117	−297 (−298, +1)	36
15months_854	1	dgRNA_4 + 7: targeted					119	−297	61
	2	dgRNA_4 + 7: targeted					119	−291 (−297, +6)	26
	3	dgRNA_4 + 7: targeted					117	−298	9
	4	dgRNA_4 + 7: targeted					117	−293 (−298, +5)	2
	5	dgRNA_4 + 7: targeted					119	−294 (−297, +3)	2
15 months_838	1	sgRNA4: large & sgRNA7: intermediate	−714	−979 (−1,000, +21)	410	−32			59
	2	dgRNA_4 + 7: targeted					117	−298	15
	3	sgRNA4: large & sgRNA7: intermediate	−715	−1,000	410	−32			10
	4	sgRNA4: intermediate & sgRNA7: small	97	−30	412	−7			8
	5	dgRNA_4 + 7: targeted					114	−298 (−301, +3)	8
15 months_3964	1	dgRNA_4 + 7: large					107	−1,326	64
	2	sgRNA 4 & 7: small^e^	119	−3	410	−6			30
	3	dgRNA_4 + 7: large					59	−1,502	6

^a^ The sgRNA4 cut site is position 118, relative to the start of exon 1.

^b^ The sgRNA7 cut site is position 415, relative to the start of exon 1.

^c^ Description of the indel(s) present in the allele based on indel size and start location. All indels are predicted to KO, bovine *NANOS3* (i.e., either a frameshift-inducing indel or an intermediate-sized indel in a protein-coding region that are predicted to generate a complete loss-of-function mutation), unless otherwise noted. Size categories: small <21 bp, intermediate = 21–500 bp, targeted = 297 ± 5 bp, large >500 bp.

^d^ Starting position of the indel, relative to the start of exon 1.

^e^ Allele #2 present in 15months_3964 has only in-frame deletions (i.e., small deletions that are multiples of 3), which results in an amino acid substitution and a deletion of 3 amino acids total.

In contrast, heifer, 15 months_854, which originally appeared to be a bi-allelic, compound heterozygote KO, was determined to be a mosaic KO carrying five unique KO alleles, each with a targeted dual gRNA_4 + 7 indels but varying by the number of bp insertions (+0 to 6 bp). Bull, 15months_838, was confirmed to be a mosaic KO with 5 unique KO alleles. The majority (59%) of his alleles had a large deletion (−979 bp) at the sgRNA4 cut site and an intermediate-sized deletion (−32 bp) at the sgRNA7 cut site. Two of the alleles (total of 23%) had the targeted dual gRNA_4 + 7 deletion and the other two alleles (18%) had a combination of small, intermediate, and large indels at both sgRNA cut sites. In total, five of the seven *NANOS3* edited bovine samples (71%) were mosaic. However, all but one of the alleles present in the mosaic samples were predicted to be KO alleles.

Moreover, the sequencing data confirmed the results that were observed when visualizing the long-range PCR productions on an agarose gel, with three of the *NANOS3* edited bovine samples (43%), 90 days_5069, 15 months_838, and 15 months_3964, carrying large (>500 bp) deletions. Interestingly, the *NANOS3* edited bull 15 months_3964 that originally appeared to carry only one allele with small, in-frame deletions was found to additionally carry two large deletion alleles (total of 70% of the reads).

Overall, the *NANOS3* long-read sequencing data confirmed many of the initial Sanger sequencing results of the short-range PCR product, but importantly it also enabled identification and measurement of the proportion of alleles present in the mosaic samples and revealed large deletion alleles (>500 bp) that ablated the short-range PCR primer sites. Ultimately, this analysis showed that a 75% (n = 6/8) total KO rate was achieved with the dgRNA_4 + 7 editing approach used in this study, and 50% (*n* = 4/8) of the samples had at least one allele with the targeted dual gRNA_4 + 7 indel (Table 3).

### 3.4 Phenotypic analysis of CRISPR/Cas9 *NANOS3* KO bovine fetal and perinatal samples

Fetuses derived from *NANOS3*-presumptively-edited embryos were collected during the stage of sexual differentiation (41 days; *n* = 2) and post sexual differentiation (90 days; *n* = 2). Additionally, gonadal samples were collected from a full-term (283 days), stillborn male calf derived from a *NANOS3*-presumptively-edited embryo. Age-matched, male, WT gonadal samples were also collected for comparisons. On average, the *NANOS3* KO fetal and perinatal testis pairs weighed less than age-matched control testis pairs, although this difference did not reach statistical significance (Figures 2B, C).

#### 3.4.1 Germline ablation observed by 41 days in CRISPR/Cas9 *NANOS3* KO bovine fetal gonads

**FIGURE 4:**
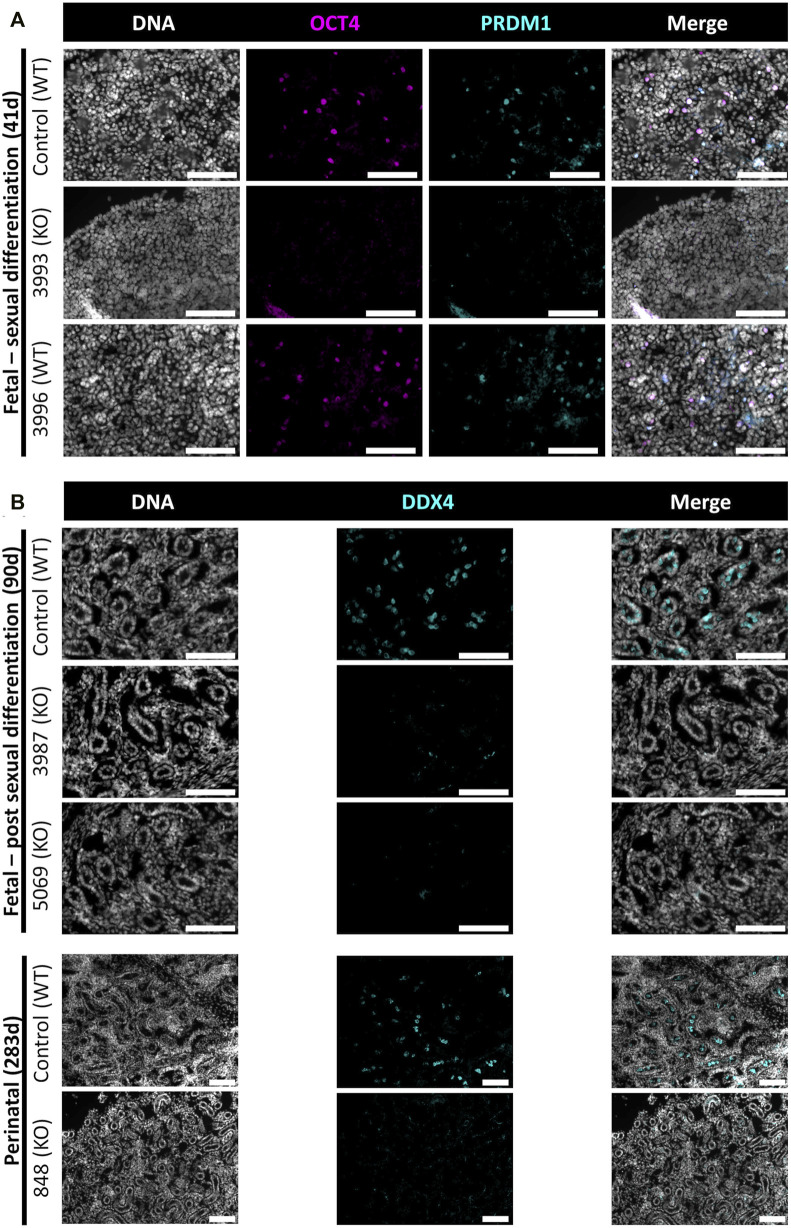
Germ cell-deficient phenotype in *NANOS3* KO fetal and perinatal testes. Representative images of immunostaining for well-conserved germ cell markers. **(A)** Immunostaining for OCT4 (magenta) and PRDM1 (cyan) in the genital ridges of samples at 41 days of fetal age. **(B)** Immunostaining for DDX4 (cyan) in the testes of samples at 90 days and 283 days of fetal age. All sections were co-stained for DNA (Hoechst 33,342; gray). Scale bars are 100 μM. *NANOS3* genotypes are noted in parentheses next to the sample name. Wildtype (WT): 100% WT, or non-mutated, genome. Knockout (KO): all alleles present in the sample were predicted to KO, or inactivate, *NANOS3* (see Figure 3; Table 3).

In this study, *NANOS3* targeted, and control fetuses were first collected at 41 days, corresponding to the stage of sexual differentiation, which is approximately 2 weeks after PGCs would first be expected to reach the genital ridge and two-three days after peak *SRY* expression. The 41 days genital ridges were co-stained for pluripotency and early PGC markers, OCT4 (also known as POU5F1) and PRDM1 (also known as Blimp1), respectively (Stukenborg et al., 2014; Planells et al., 2019; Soto and Ross, 2021). The 41 days control samples and the unedited (100% WT) 41 days_3996 sample stained positive for pluripotency and early PGC markers, OCT4 and PRDM1, respectively (Figure 4A). In contrast, the genital ridge of sample 42 days_3993, which was determined to be a *NANOS3* KO, stained negatively for both OCT4 and PRDM1, showing germline ablation at this stage (Figure 4A).

The 90 and 283 days testes samples were stained for a known germ cell marker, DDX4 (also known as “Vasa”), which is expressed in differentiated germ cells from spermatogonia to round spermatids (Raz, 2000; Pennetier et al., 2004; Bartholomew and Parks, 2007; Caires et al., 2009; Stukenborg et al., 2014; Planells et al., 2019). After sexual differentiation, testis cord formation was observed in both the control and *NANOS3* KO fetal testes at 90 days (Figure 4B). However, staining for the germ cell marker, DDX4, was only observed in the developing testis cords of control samples. Additionally, seminiferous cord development was observed in both the control and *NANOS3* KO perinatal (283 days) testes (Figure 4B). In the perinatal control testes, DDX4 positive cells were observed in the center of many of the seminiferous cords. In contrast, no DDX4 positive cells were observed in the *NANOS3* KO sample, 283 days_848, even though the seminiferous cord structures were present. Overall, the *NANOS3* KO testes had similar testis cord formation patterns compared to age-matched control testes, indicating that the somatic support cells remained intact through fetal development in the *NANOS3* KO testes, even in the absence of germ cells (Figures 4A, B).

#### 3.4.2 CRISPR/Cas9 *NANOS3* KO bovine fetal and perinatal testes showed specific germline ablation with intact somatic support cell populations

**FIGURE 5:**
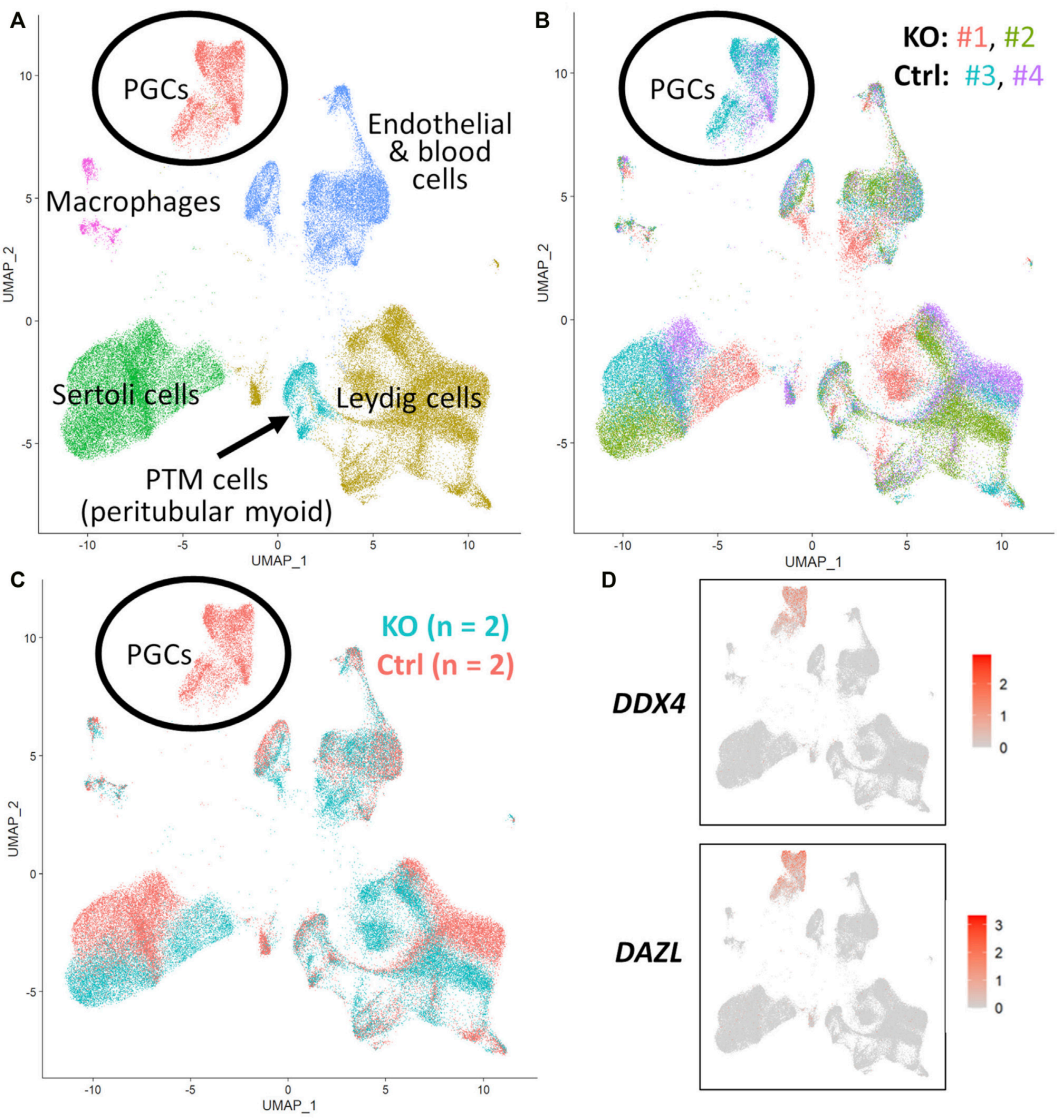
scRNA-Seq analysis of 90 days fetal testes, comparing *NANOS3* KO samples (*n* = 2) to control (ctrl) samples (*n* = 2). **(A)** UMAP plot of different cell populations of the fetal testis. Clusters were identified based on expression of known marker genes. **(B)** UMAP plot colored by individual samples (*n* = 4). **(C)** UMAP plot colored by treatment showing that only control samples are present in the primordial germ cell (PGC) cluster. **(D)** UMAPs showing differential expression of known late PGC/gonocyte markers, *DAZL* and *DDX4*.

**FIGURE 6:**
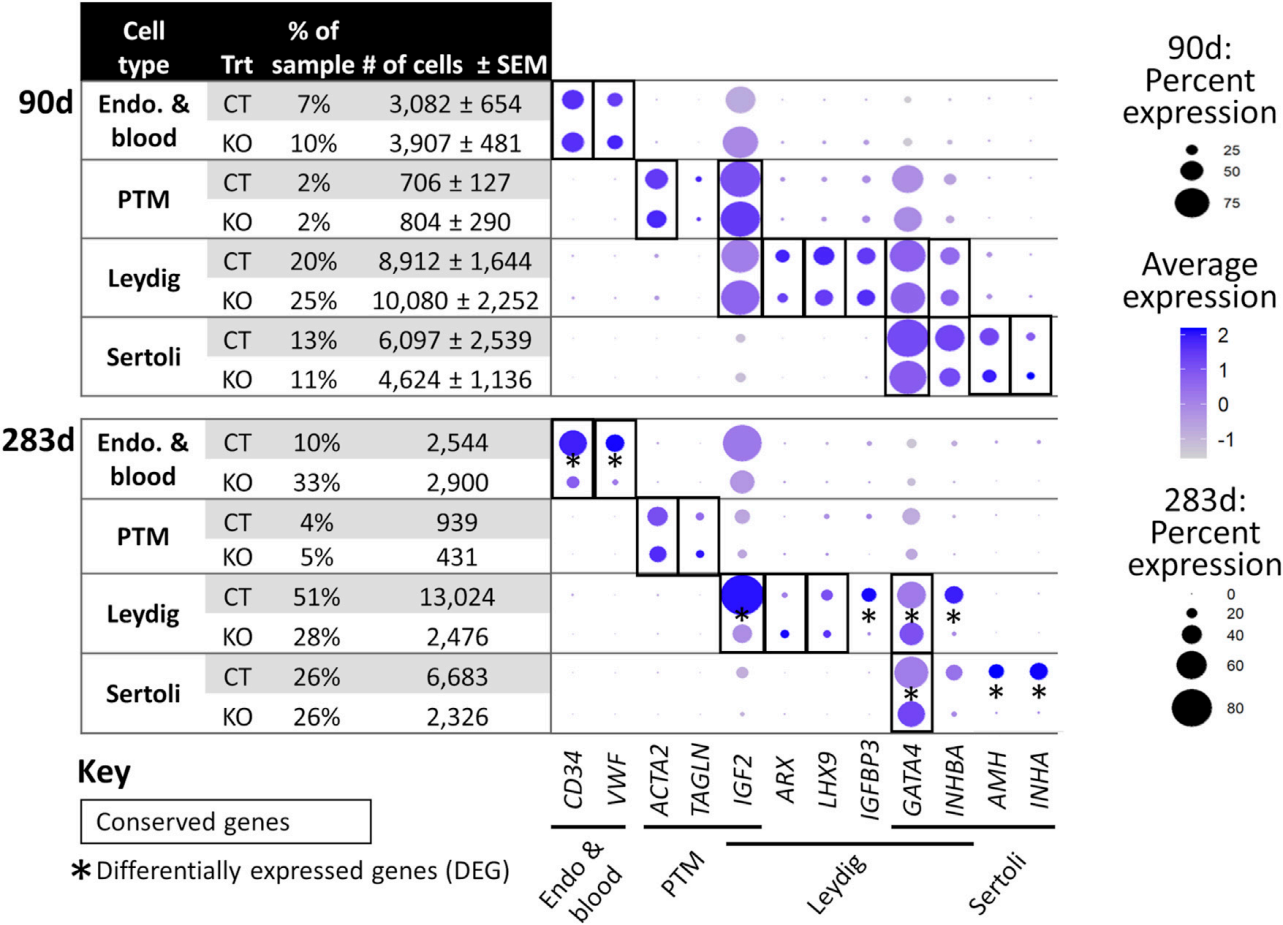
scRNA-Seq differential gene expression analysis of *NANOS3* KO and WT control (CT) testes at 90 days (top panel) and 283 days (bottom panel) of fetal age. Known-marker genes for different testicular somatic cell populations are listed across the bottom of the dot plots. Some genes are shared makers for 2 cell populations. The dot size represents the percent of cells present in a particular cell type cluster expressing a particular gene (larger dots indicate a greater proportion of cells). The color of the dot represents the average scaled expression level of a particular gene in a particular cell type cluster (darker indicates higher average expression). Conserved genes (black boxes) were defined as those genes that were more highly expressed (log-transformed fold-change ≥0.5) in a specific cell type of both treatments when compared to all other cell types at that timepoint. Differentially expressed genes (DEG; black asterisks) had significantly (*p* ≤ 0.05) different expression (log-transformed fold-change ≥0.5) between treatments. Some genes were both conserved and DEG. Abbreviations: Endothelial (Endo.), Peritubular Myoid (PTM), treatment (Trt), Hematopoietic Progenitor Cell Antigen CD34 (*CD34*), Von Willebrand Factor (*VWF*), Actin Alpha 2 Smooth Muscle (*ACTA2*), Transgelin (*TAGLN*), Insulin Like Growth Factor 2 (*IGF2*), Aristaless Related Homeobox (*ARX*), LIM Homeobox 9 (*LHX9*), Insulin Like Growth Factor Binding Protein 3 (*IGFBP3*), GATA Binding Protein 4 (*GATA4*), Inhibin Subunit Beta A (*INHBA*), Anti-Müllerian Hormone (*AMH*), and Inhibin Subunit Alpha (*INHA*).

**FIGURE 7:**
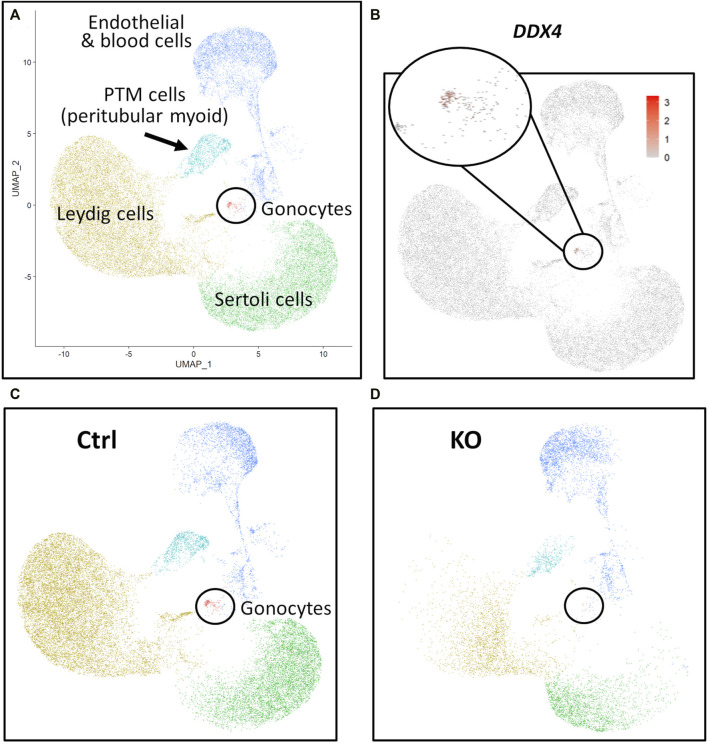
scRNA-Seq analysis of 283 days perinatal testes, comparing a *NANOS3* KO sample to a control (Ctrl) sample. **(A)** UMAP plot of different cell populations of the perinatal testis. Clusters were identified based on expression of known marker genes. **(B)** UMAP showing differential expression of known gonocyte marker *DDX4*. **(C, D)** UMAP plots colored by cell type for Ctrl **(C)** and KO **(D)** cells, showing the lack of gonocytes (i.e., absence of red dots in the black circle) in the *NANOS3* KO sample.

scRNA-Seq analysis was employed to confirm immunostaining results and fully characterize the germ and somatic cell populations of the 90 and 283 days *NANOS3* KO, compared to WT, bovine testes (Figures 5–7). Each timepoint was analyzed individually.

For the 90 days timepoint, a total of 45,630 and 40,237 cells passed quality filtering and were analyzed for control (*n* = 2) and KO (n = 2) treatments, respectively. On average, approximately 21,000 cells were analyzed for each sample (control_1 = 25,909, control_2 = 19,721, KO_3987 = 15,799, and KO_5069 = 24,438). Clusters were identified as cell types based on differential gene expression of well-known marker genes. The 90 days PGC cluster was identified by high expression of late PGC/gonocyte markers, *DDX4* and *DAZL* (Figure 5D). At 90 days pluripotency (e.g., *OCT4* and *NANOG*) and early PGC (e.g., *NANOS3*) markers were only expressed in a small number of PGCs, indicating that at 90 days the majority of PGCs are in the late PGC/gonocyte stage. The early PGC marker, *KIT*, was expressed highly in the 90 days PGC cluster, but also highly expressed in the endothelial and blood cells cluster, which agrees with several studies showing that *KIT* is not a specific method to identify germ cells (Ohno and Gropp, 1965; Lavoir et al., 1994; Kritzenberger and Wrobel, 2004; Soto and Ross, 2021). The identified PGC cluster exclusively contained control cells, with no cells from the KO samples present (Figure 5C). Additionally, there was no expression of *NANOS3* or late PGC markers, *DDX4* or *DAZL*, in the *NANOS3* KO testicular cells. In contrast, *NANOS3* was expressed in 2% of the control PGCs, and the PGC cluster represented 9% of the total control cells analyzed. Key somatic support cell populations, including Sertoli, Leydig, and Peritubular Myoid (PTM) cells, were identified in all four samples (Figures 5A, B). Additionally, the 90 days KO somatic cell populations were present in similar proportions to the WT control samples, and the majority of marker genes for the somatic cell populations were conserved across treatments (Figure 6, 90 days). Conserved genes were defined as those genes that were differentially expressed (log-transformed fold-change ≥0.5) in a specific cell type of both treatments when compared to all other cell types at the same timepoint.

For the 283 days timepoint, a total of 25,733 and 8,828 cells passed quality filtering and were analyzed for the control and KO samples, respectively. Clusters were identified as cell types based on differential gene expression of well-known marker genes. Similar to the 90 days samples, the 283 days germ cell cluster was identified by high expression of gonocyte markers, *DDX4* and *DAZL* (Figure 7B). There was no *NANOS3* expression observed in the *NANOS3* KO sample*.* Only control cells were present in the gonocyte cluster (Figure 7D) and less than 1% of the control cells expressed *NANOS3* (Figure 7C). At 283 days, the gonocytes represented a much smaller proportion (1%) of the total control cells analyzed than compared to 90 days. Key somatic support cell populations, including Sertoli, Leydig, and PTM cells, were identified in both samples (Figure 7A), but there was a smaller proportion of Leydig cells present in the KO (28%) compared to the control (51%) (Figure 6, 283 days). Several key marker genes for the somatic cell populations were conserved across treatments. However, many of these marker genes also had significantly (*p* ≤ 0.05) different expression (log-transformed fold-change ≥0.5) between treatments (Figure 1D). Ultimately, the scRNA-seq analysis showed a complete loss of PGCs and gonocytes in *NANOS3* KO fetal and perinatal testes, while maintaining the development of somatic support cells.

### 3.5 Phenotypic analysis of CRISPR/Cas9 *NANOS3* edited cattle

**FIGURE 8:**
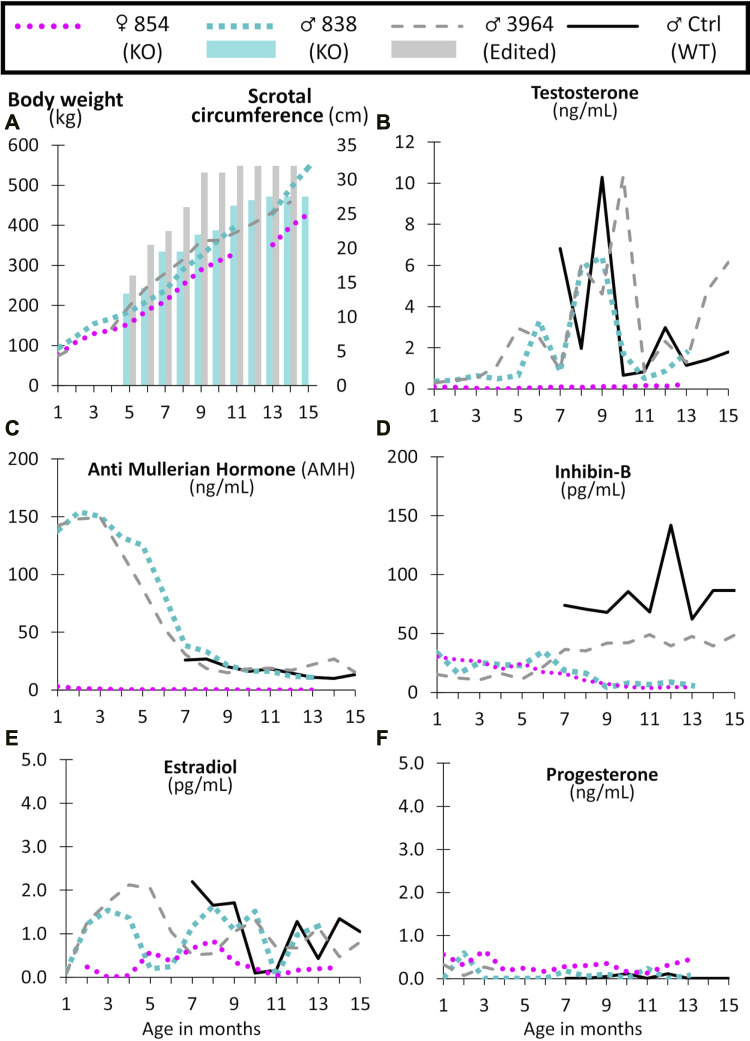
Monthly body weight (left axis), scrotal circumference (right axis) **(A)**, and reproductive hormone levels **(B–F)** of CRISPR/Cas9 *NANOS3* edited cattle from 1 to 15 months old. Monthly serum hormone levels for male primary hormones, testosterone (ng/mL), AMH (ng/mL), and inhibin-B (pg/mL), and female primary hormones, estradiol (pg/mL) and progesterone (ng/mL). Sample key: *NANOS3* KO heifer #854 (pink round dots), *NANOS3* KO bull #838 (blue square dashes and blue bar for SC), *NANOS3* edited bull #3964 (grey dashes and grey bar for SC), genetic wildtype (WT) control (ctrl) bull (black solid line).

The reproductive development and capabilities of the live *NANOS3* edited cattle (*n* = 3; two males and one female) were characterized through post-pubertal age (15 months). Monthly, body weights and male scrotal circumferences were measured. Additionally, blood was collected monthly for hormone analysis from the live *NANOS3* edited animals (*n* = 3) and a WT bull (*n* = 1). Body weights followed a normal linear growth pattern (Figure 8A).

#### 3.5.1 Reproductive hormone levels of CRISPR/Cas9 *NANOS3* edited cattle

##### 3.5.1.1 Male hormones

Both the *NANOS3* KO #838 and edited #3964 bulls had increased testosterone secretion with age, exceeding 1 ng/ml after approximately 4-months-of-age, and then reaching peak levels (6 ng/ml and 10 ng/ml, respectively) during puberty (7–9 months old; Figure 8B). The control bull also reached a peak testosterone level of 10 ng/ml during puberty. In contrast, testosterone was present at low levels (average 0.1 ng/ml) in the *NANOS3* KO heifer (#854) and never exceeded 0.3 ng/ml.

In the *NANOS3* KO #838 and edited #3964 bull samples, AMH levels peaked during the first few months of life (reaching 150 ng/ml) then decreased to stable levels during and post-puberty (Figure 8C). As for the control bull, since sampling began at 7 months old, his peak level was unknown, but his post-pubertal AMH levels were similar to those of both *NANOS3* targeted bulls, ranging from 10 to 30 ng/ml. The *NANOS3* KO heifer #854 had only 3 ng/ml AMH at 1 month old, decreasing to undetectable levels (<0.01 ng/ml) by 10 months old.

Both the *NANOS3* KO #838 and edited #3964 bulls had low pre-puberty levels of inhibin-B (10–35 pg/ml) (Figure 8D). When puberty started, around 7 months old, inhibin-B levels in the *NANOS3* KO #838 bull decreased to less than 10 pg/ml by 9 months of age. In contrast, the *NANOS3* edited #3964 bull’s inhibin-B levels increased to 40–50 pg/ml during puberty and post-puberty. During the same time (7–15 months old), the average inhibin-B level for the control bull was 82 pg/ml. The *NANOS3* KO heifer #854, had a similar pattern to the *NANOS3* KO bull, with low pre-pubertal levels (15–30 pg/ml) which decreased to below 10 pg/ml after 7 months of age.

##### 3.5.1.2 Female hormones

The *NANOS3* KO heifer #854 had an average estradiol level of 0.5 pg/ml, with levels post-puberty never exceeding 0.9 ng/ml (Figure 8E). Both the *NANOS3* KO #838 and edited #3964 bulls had similar average estradiol levels from 2 to 4 months old, 1.4 pg/ml and 1.7 pg/ml, respectively. Additionally, during puberty and post-puberty all three bulls, including the control bull, had similar estradiol levels of approximately 1 pg/ml. Post-pubertal progesterone levels in the *NANOS3* KO heifer (#854) never exceeded 0.4 ng/ml and progesterone was detected at low levels (<0.5 ng/ml) in all of the bulls in this study (Figure 8F).

#### 3.5.2 Germline ablation in CRISPR/Cas9 *NANOS3* KO bull #838

**FIGURE 9:**
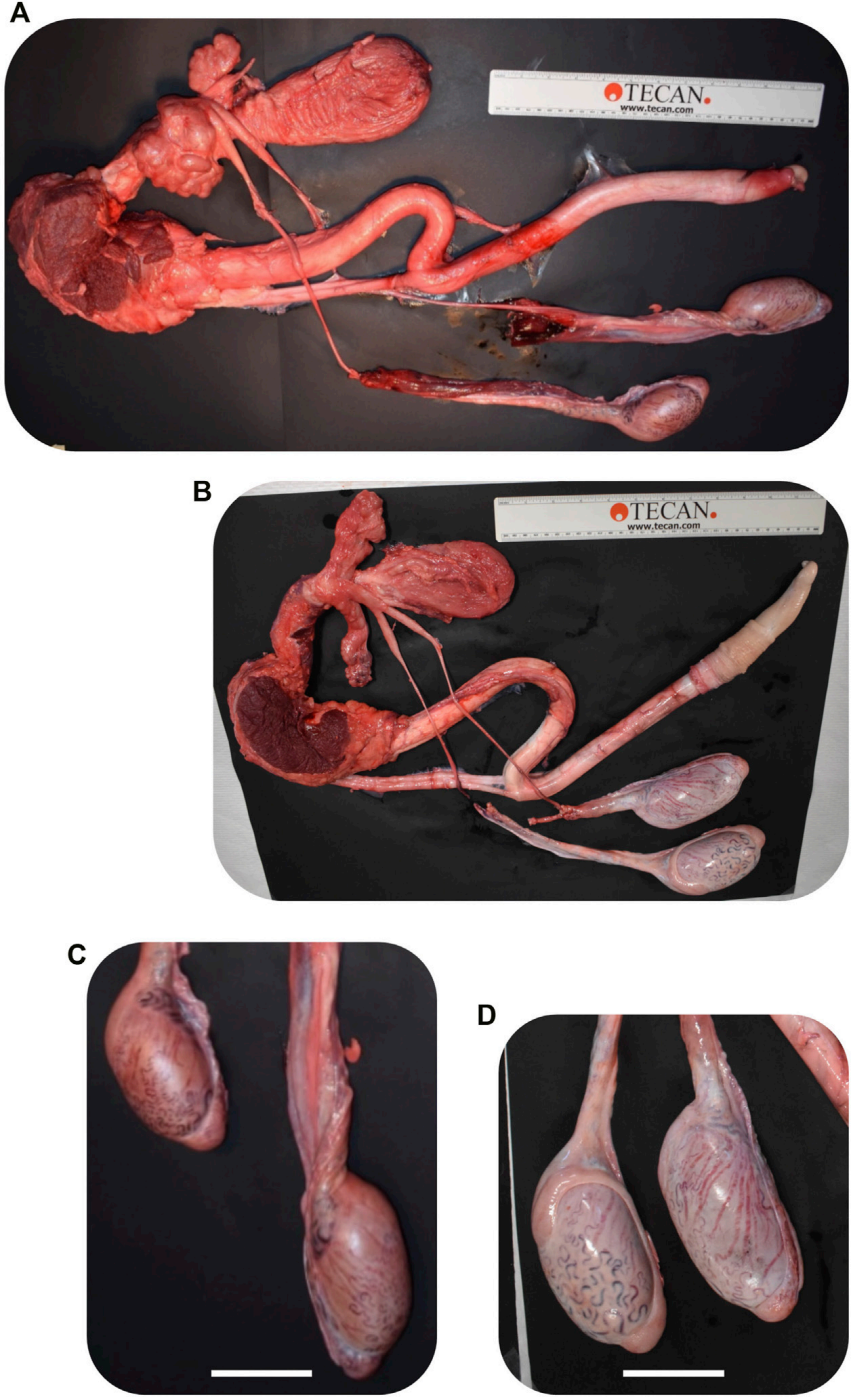
Comparison of CRISPR/Cas9 *NANOS3* KO bull #838 **(A, C)** and edited bull #3964s **(B, D)** reproductive tracts **(A, B)** and testes size **(C, D)** at 15 months of age. Scale bars are 5 cm. Ruler is 31.5 cm.

**FIGURE 10:**
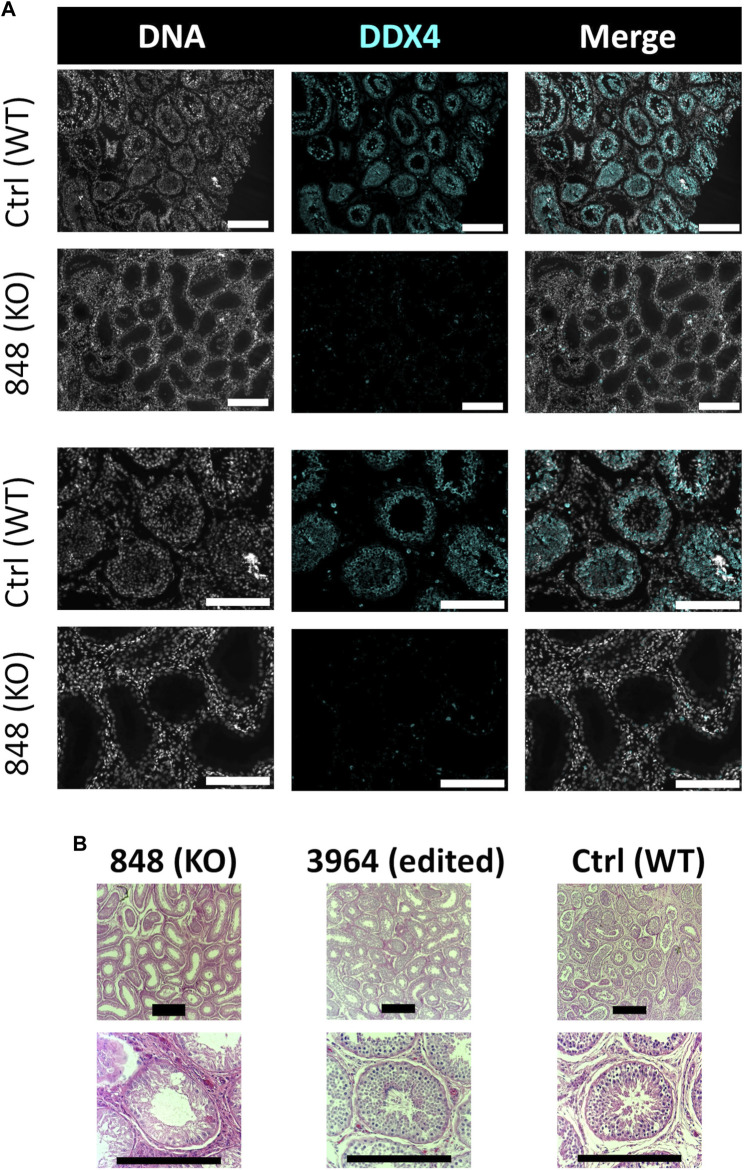
Histological analysis of CRISPR/Cas9 *NANOS3* KO bull #838 and edited bull #3964s testes. **(A)** Representative images of DDX4 immunostained (cyan) testis cross-sections from KO bull #838 compared to an age matched, wildtype (WT) bull. All immunostained sections were co-stained for DNA (Hoechst 33,342; gray). **(B)** Representative images of H&E-stained testis cross-sections from KO bull #838, edited bull #3964, and an age matched, WT bull. Scale bars are 100 μM. Histology indicates that all samples have Sertoli cells lining the seminiferous tubules, but KO bull #838 lacks any spermatogenesis.

Bull #838 was a *NANOS3* mosaic KO (Table 3) and therefore it was hypothesized that there would be a complete loss of germ cells in bull #838, but otherwise normal gonadal development (Tsuda et al., 2003). At 12 months of age, bull #838 had a masculine appearance, demonstrated normal libido, an anatomically normal reproductive tract and normal testicular development, although his scrotal circumference (27 cm) was smaller than expected for age and breed matched controls. However, microscopic evaluation of an ejaculate obtained *via* electroejaculation revealed seminal plasma only with no spermatozoa present. These results were repeated and confirmed with BSEs at 13 and 15 months old. At 15 months old, bull #838 was harvested and his reproductive tract was collected. Bull #838s reproductive tract was confirmed to be anatomically normal with all accessory sex glands present (Figures 9A, C). Additionally, cross-sections of bull #838s testes were immunostained for the germ-cell marker, DDX4 (Figure 10A) and stained with H&E (Figure 10B). Compared to an age matched, WT (*NANOS3* ^+/+^) bull, bull #838 lacked any spermatogenesis, but importantly had Sertoli cells lining the seminiferous tubules.

#### 3.5.3 Intact germline in CRISPR/Cas9 *NANOS3* edited bull #3964

Bull #3964 was carrying three mutated alleles and zero WT alleles (Table 3). However, one allele (30% of reads) had only small, in-frame deletions that were all outside of the highly-conserved N-terminal and zinc finger binding domains and resulted in only a few amino acid changes (Table 3; Figures 3C, D). Therefore, it was possible that this in-frame deletion allele could result in a functional *NANOS3* protein and thus an intact germline. At 12 months of age, bull #3964 had a masculine appearance, demonstrated normal libido, and passed a BSE. Bull #3964 had an anatomically normal reproductive tract, normal testicular development with adequate scrotal circumference (32 cm), and produced a satisfactory ejaculate for his age (30% motility, 78% normal cells, 11% head abnormalities, 11% midpiece abnormalities, 0% tail abnormalities). At 15 months bull #3964 was harvested and his reproductive tract was collected. Bull #3964s reproductive tract was confirmed to be anatomically normal with all accessory sex glands present and an adequate scrotal circumference of 32 cm (Figures 9B, D). Additionally, spermatogenesis was evident in bull #3964s testes *via* H&E staining (Figure 10B).

#### 3.5.4 Germline ablation in CRISPR/Cas9 *NANOS3* KO Heifer #854

**FIGURE 11:**
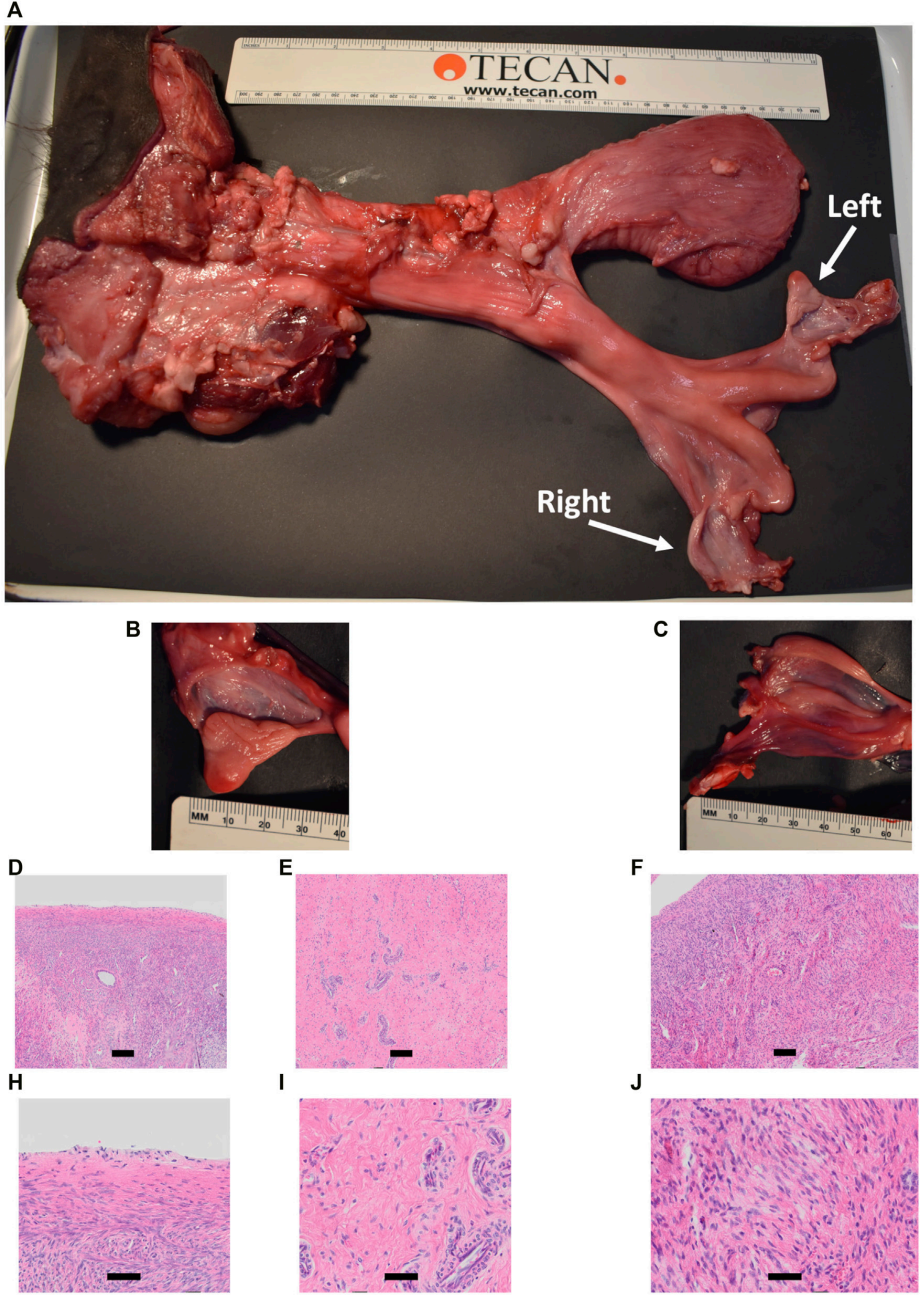
Phenotypic analysis of CRISPR/Cas9 *NANOS3* KO Heifer #854 at 15 months of age. **(A)** Heifer #854s reproductive tract. **(B, C)** Heifer #854s left ovary **(B)** and right putative primitive streak **(C)**. **(D–J)** Representative images of H&E-stained ovary cross-sections from heifer #854 showing a complete lack of oogenesis.

Heifer #854 was a mosaic KO, with five unique alleles that all had targeted dual gRNA_4 + 7 indels (291–298 bp; Table 3) and zero WT alleles. Due to these KO mutations, it was hypothesized that there would be a complete loss of germ cells in heifer #854, but otherwise normal gonadal development (Tsuda et al., 2003; Ideta et al., 2016). Heifer #854 had a feminine appearance, and her behavior was observed through puberty until 15 months but visual signs of estrus were never observed. UC Davis veterinarians performed a reproductive exam on heifer #854, around 14 months of age. A small, involuted, and hypoplastic reproductive tract, with a small cervix and flaccid uterine horns, were observed *via* palpation, which are similar characteristics of a juvenile or freemartin female. Using ultrasound, the right ovary was unable to be imaged and the left ovary was observed to be small (<1 cm) with no identifiable structures or observable follicular development. At 15 months old, heifer #854 was harvested and her reproductive tract was observed to be anatomically abnormal, with a small clitoris, long anterior vagina, and a putative primitive streak in place of the right ovary (Figures 11A–C). Additionally, cross-sections of the left ovary and right primitive streak were processed for H&E analysis, which showed a complete lack of oogenesis (Figures 11D–J).

#### 3.5.5 Meat composition values of CRISPR/Cas9 *NANOS3* KO cattle were within normal variation

The average meat composition values for proximate analysis and minerals of the *NANOS3* KO heifer #854 and bull #838 were within the normal variation seen in international meat composition databases (Table 4) (Trott et al., 2022).

**TABLE 4:** Meat compositions analysis of sirloin cap and chuck arm from CRISPR/Cas9 *NANOS3* KO cattle (*n* = 2) and values from Trott et al. (2022) analysis of international nutrient databases.

	NANOS3 KO cattle average (SD)	Literature mean^a^	Literature range^a^	Within literature range?
Proximate analysis				
Ash (%)	0.85 (0.06)	1.02	0.1–2.62	Yes
Protein (%)	22.175 (1.53)	20.5	11.0–29.8	Yes
Crude fat (%)	1.75 (1.22)	8.64	0.5–42.5	Yes
Minerals				
Iron (%)	0.002 (0.0005)	0.0021	0.0007–0.005	Yes
Phosphorus (%)	0.1965 (0.01)	0.185	0.09–0.37	Yes
Zinc (ppm)	45.25 (9.12)	47.1	10–98.5	Yes

^**a**^ Values from Trott et al. (2022) analysis of international nutrient databases.

## 4 Discussion

In this study, direct cytoplasmic microinjection of the CRISPR/Cas9 system into *in vitro* produced bovine embryos was optimized to KO *NANOS3* with high efficiency and repeatability. Using these methods three live *NANOS3* KO cattle were produced and the effect of eliminating *NANOS3* on bovine germline development was characterized from fetal development through reproductive age.

The gene KO approach used in this study, involving co-injection of two selected sgRNA/Cas9 RNP complexes into bovine zygotes (6 hpf), resulted in a high *NANOS3* KO rate in developing embryos (79%, *n* = 22/28, 4 replicates), while maintaining an acceptable blastocyst development rate (19%, n = 28/150, 4 replicates). Bovine *NANOS3* is a 2,633 bp gene with two exons (Figures 1B, C), and exon 1 (451 bp) contains the highly conserved coding regions for the N-terminal and zinc finger binding domains (Beer and Draper, 2013; Suzuki et al., 2014), thus exon one was targeted in this study. A dual gRNA approach was used and sgRNAs #4 and #7 (dual gRNA_4 + 7) were selected based on their mutation efficiencies and genomic locations to target both the 5′ and 3’ regions of exon 1, which, when acting together, should completely remove the critical zinc finger binding domain (297 bp between sgRNA cut sites; Figures 1C–E).

For the eight bovine samples, initial *NANOS3* genotype analysis via short-range PCR amplification and Sanger sequencing of the *NANOS3* exon one target region, confirmed the high mutation efficiency of the dual gRNA_4 + 7 approach used in this study. Seven of the eight *NANOS3* targeted bovine samples (87.5%) were successfully edited (0% WT alleles remained). Additionally, four of the edited samples (57%) had at least one allele with a targeted dual gRNA_4 + 7 indel. All but one allele present in the edited *NANOS3* bovine samples were predicted to be KO alleles. In these experiments, a KO allele was defined as having either a frameshift-inducing indel or an intermediate sized indel (>21 bp) in a protein-coding region that were predicted to generate a complete loss-of-function mutation.

The one allele that was predicted not to be a KO was allele #2 (Table 3) in the edited bull #3964 that had two small, in-frame deletions. The allele resulted in one amino acid substitution and a deletion of three total amino acids, but each of the mutations were outside of the highly conserved N-terminal and zinc finger domains (Figures 3C, D). The evolutionarily conserved zinc finger domain, which consists of two consecutive CCHC-type zinc finger motifs, is indispensable for *in vivo* functions in *Drosophila* where the *Nanos* gene was first identified (Wang and Lehmann, 1991). Additionally, studies in mice have shown that both an intact N-terminal region and zinc finger domain are essential for murine *Nanos2* functions *in vivo*, but other regions may be dispensable (Suzuki et al., 2012; Suzuki et al., 2014). The exact amino acid sequence predicted to result from the mutated allele in 15 months_3964 was not found in any other species when a protein BLAST analysis was conducted, and it was unknown if the deleted amino acids were necessary for *NANOS3* protein function. Therefore, no *a priori* information was available as to whether this in-frame allele could be functional.

In this initial genotype analysis, many samples appeared to be mosaic but given the limitations of Sanger sequencing, the proportion of different alleles present in each sample could not be accurately discerned. Additionally, several studies have reported that large deletions of up to several thousand bases occur with high frequencies (up to 15%) at the Cas9 on-target cut sites. Therefore, further sequence analysis was completed for all eight CRISPR/Cas9 *NANOS3*-presumptively-edited bovine samples to 1) identify and measure the proportion of alleles present in the mosaic samples, and 2) detect potential large deletions present in the samples. Using long-range PCR (6,274 bp), three of the samples (43%) were found to have previously undetected alleles with large (960–1,502 bp) deletions (Figure 3B; Table 3). These deletions eliminated the short-range PCR primer binding sites commonly used for on-target analysis. Consequently, these alleles would have remained undetected using traditional screening methods. These findings align with studies that highlight the occurrence of both small indels and large deletions following Cas9 cleavage, emphasizing the importance of evaluating the presence of large deletions in genome editing experiments (Park et al., 2022). Interestingly, in the *NANOS3* edited bull #3964, the long-read sequencing revealed that the in-frame allele represented only 30% of the reads, while the remaining 70% of the reads from this sample were comprised of two large deletion alleles, each predicted to be non-functional. Overall, a 71% mosaicism rate (n = 5/7) was observed in the *NANOS3* edited bovine samples. However, it is noteworthy that all but one of the 23 edited alleles resulted in a predicted KO or loss of function.

Murine studies have shown that *Nanos3* mutations do not affect PGC specification but rather impair the subsequent survival of PGCs during their migration to the developing gonad. In *Nanos3* KO murine fetuses a small proportion of PGCs (approximately 1/5 of the original population) can reach the genital ridge, but these cells are quickly lost to apoptosis and by murine day 10 no PGCs remain in the bipotential gonad (Tsuda et al., 2003; Suzuki et al., 2007). These studies were foundational to demonstrate that mammalian *Nanos3* maintains the germ cell lineage by suppressing apoptotic pathways (Suzuki et al., 2008). *NANOS3* KO pig fetuses were also found to have significantly fewer migrating PGCs on day 18 of fetal age and subsequently a complete loss of PGCs in both fetal ovaries and testis after gonadal sex differentiation, day 35 (Park et al., 2023).

In cattle, putative migrating PGCs can first be identified at 18 days of fetal age and the first PGCs reach the developing genital ridge around 27 days of fetal age, with the majority of PGCs arriving by 31 days of fetal age (Wrobel and Süß, 1998). In male cattle, *SRY* is first expressed at 35 days of fetal age and sexual determination occurs from day 38–39 of fetal age when SRY expression peaks (Wrobel and Süß, 1998; Ross et al., 2009; Planells et al., 2019). Testis cords become distinguishable during early gonad differentiation around 42–44 days of fetal age (Ross et al., 2009; Planells et al., 2019). Given the different developmental timing of bovine development compared with murine and porcine, it was previously unknown if or how early bovine PGCs would be eliminated in *NANOS3* KO gonads. The previous *NANOS3* KO study in female cattle provided valuable evidence that *NANOS3* plays a similar role in cattle as it does in mice and pigs (Ideta et al., 2016). However, this study only produced one *NANOS3* KO female fetus that was collected at 190 days of development, which is past the PGC stage.

In this current study, *NANOS3*-targeted and control fetuses were examined at 41 days, which corresponds to the stage of sexual differentiation. At this point, it is expected that PGCs would have reached the genital ridge (Wrobel and Süß, 1998; Stukenborg et al., 2014; Planells et al., 2019; Soto and Ross, 2021). Consistent with this, the 41 days control sample and the unedited (100% WT) 41 days_3996 sample showed the presence of PGCs in the genital ridge, as observed through immunofluorescence analysis. However, in the *NANOS3* KO sample (42 days_3993), no PGCs were observed in the genital ridge. This indicates that in male *NANOS3* KO gonads, bovine PGCs were eliminated as early as 41 days. Although it is possible that as seen in mice and pigs, bovine PGCs could have reached the genital ridge in *NANOS3* KO cattle, importantly they were eliminated by the bipotential gonad stage.

During bovine fetal development, following gonad sexual differentiation (approximately 44 days of fetal age), late PGCs, known as gonocytes, begin to cluster together with developing Sertoli cells surrounding them. This clustering leads to the formation of testicular cords, and as fetal development progresses, distinct seminiferous cords are formed with fully enclosed populations of gonocytes by pre-Sertoli cells. At birth, these gonocytes are separated from the basement membrane of the seminiferous cords by immature Sertoli cells (Skinner and Anway, 2005; Ross et al., 2009; Culty, 2013; Planells et al., 2019). While this pattern of testis cord formation was observed in both the control and *NANOS3* KO fetal and perinatal testes, gonocytes were only observed in the developing testis cords of control samples. This finding is consistent with the absence of PGCs in the 41 days *NANOS3* KO gonads. Notably, despite the lack of germ cells in the *NANOS3* KO testes, the somatic support cells, such as Sertoli cells, remained intact.

Additionally, the scRNA-Seq analysis of the 90 days and 283 days *NANOS3* KO testes confirmed the immunofluorescence results, by showing the presence of key somatic support cell populations (e.g., Sertoli cells, Leydig cells, and PTM cells), but a complete loss of PGCs and germ cells. Importantly, no *NANOS3* expression was observed in the *NANOS3* KO samples at either timepoint, although *NANOS3* was only expressed by a small proportion of 90 days PGCs (2%) and 283 days gonocytes (0.7%) in the control samples. Prior RNA-Sequencing studies of cattle gonadal samples documented *NANOS3* expression in both males and females from 35 to 43 days of fetal age (Planells et al., 2019). Additionally, scRNA-Seq analysis of bovine ovaries around 50 days of fetal age found that the majority of PGCs were in the early stage of differentiation, as the majority of cells expressed at least one pluripotency and one early PGC marker, including 80% expressing *NANOS3* (Soto and Ross, 2021). A smaller proportion of 50-day female fetal PGCs were also expressing late PGC markers, so it appeared that a subset of cells were already transitioning toward a more advanced stage at that timepoint (Soto and Ross, 2021). In this current study which looked at further developed fetuses (90 and 283 days), only a small proportion of PGCs and gonocytes, respectively, expressed *NANOS3* in the control samples, which aligns with *NANOS3* being a known marker of early stage PGCs. From 90 days to 283 days of fetal age, the proportion of germ cells present in the WT control testes decreased from 9% to 1%. The 283 days *NANOS3* KO sample had significantly fewer cells analyzed (8,000) compared to all other samples at both timepoints (average of approximately 20,000 per sample), so it is possible that the lack of germ cells observed in this sample was due to the limited number of cells analyzed. However, immunofluorescence analysis of testicular cross-sections from this same 283 days *NANOS3* KO sample also showed a complete lack of germ cells, thus supporting the scRNA-Seq analysis finding.

In the *NANOS3* KO testes, even in the absence of germ cells, many of the known marker genes for the somatic cell populations shared similar expression patterns across treatments at both timepoints. In the 90 days KO testes, the somatic cell populations were also present in similar proportions to the WT control samples. In contrast, in the 283 days KO testis there were 23% fewer Leydig cells compared to the WT control sample and many of the known marker genes for somatic cell populations had significantly (*p* ≤ 0.05) different expression levels (log-transformed fold-change ≥0.5) between treatments. Given the strong relationship and constant communication between germ and somatic cells during gonad development, these differences found at 283 days could be due to the lack of germ cells impairing the somatic gonad development. However, in the 90 days samples there was large variation in cell numbers per cell type between biological replicates of both treatments. Since there were no biological replicates at the 283 days timepoint, it is unknown if the differences in somatic cell populations were due to the *NANOS3* KO or individual sample variation. Importantly, histological analysis of testicular cross-sections from this same 283 days *NANOS3* KO sample showed similar testis cord formation patterns compared to an age-matched control testis. Taken together, these findings indicate that the somatic support cell structures remained intact through bovine fetal development, but these cells may have had impaired communication and endocrinological functions later in development due to the lack of germ cells in *NANOS3* KO gonads.

To the best of our knowledge, this is the first study to produce live *NANOS3* KO cattle, which allowed for the evaluation of the effect of disrupting *NANOS3* on reproductive development in cattle from birth through puberty and into reproductive maturity. Overall, this study demonstrated that bovine *NANOS3* is necessary for both male and female bovine germline development.

*Nanos3* KO male mice have been reported to have atrophic testes, yet have intact seminiferous tubules, with no detectable spermatozoa (Tsuda et al., 2003; Suzuki et al., 2008; Miura et al., 2021). Additionally, *NANOS3* KO boars at 3 months old (pre-puberty) and at 6 months old (during puberty) also had no detectable germ cells, but had intact seminiferous tubules (Kogasaka et al., 2022). In this study, the *NANOS3* mosaic KO bull #838 was found to copy the phenotype of *Nanos3* KO male mice and boars. As expected, the *NANOS3* KO bull was germline ablated as evidenced by the lack of spermatozoa in his ejaculate and his germ cell deficient testis (Figures 10A, B). Additionally, he had an anatomically normal reproductive tract, with all accessory sex glands present and his testis had intact seminiferous tubules. However, his 15 months scrotal circumference (27.5 cm) was below the industry BSE benchmark of a minimum scrotal circumference of 32 cm for bulls 15–18 months old. Interestingly, the adult size of the *NANOS3* KO testes was less affected in bulls (86% of WT size) than in mice (20%–30% of WT size) (Tsuda et al., 2003; Miura et al., 2021). Moreover, bull #838 demonstrated normal libido and his serum hormone levels for key reproductive hormones were within the normal ranges throughout development, apart from his post-puberty inhibin-B levels. Inhibin-B is produced predominantly by Sertoli cells. However, in mammals, it is hypothesized that the combination of Sertoli cell proliferation along with germ cell complement contributes to the overall production level of inhibin-B. For instance, men with azoospermia (i.e., complete absence of spermatozoa in the ejaculate) have been reported to have low serum inhibin-B levels (Brugo-Olmedo et al., 2001; Stewart and Turner, 2005). Therefore, in the germline-ablated bull #838, it is logical to observe low levels of inhibin-B during post-puberty, as there were no germ cells present to interact with the Sertoli cells to modulate inhibin-B production. Overall, phenotypic analyses indicated that bull #838 had an intact and activated hypothalamic-pituitary-gonadal (HPG) axis, went through puberty, had functional testicular interstitial tissue, and at least prior to puberty, had endocrinologically functional Sertoli cells.

The fertile CRISPR/Cas9 *NANOS3* edited bull #3964 suggests that bovine *NANOS3* is a haplosufficient gene. Although bull #3964 was carrying three mutated alleles, including two alleles with large (>500 bp) deletions, and zero WT alleles, the one in-frame allele (30% of reads) appears to have resulted in a functional *NANOS3* protein that was sufficient for male germline development (Figures 3C, D; Table 3). This finding aligns with the observation that both male and female heterozygous *Nanos3* ^+/−^ mice are fertile with morphologically and functionally normal gonads (Tsuda et al., 2003).

Ovaries from post-pubertal, *NANOS3* KO, female mice and pigs exhibit diminished size and a lack of oogenesis compared to control, WT ovaries (Tsuda et al., 2003; Suzuki et al., 2008; Kogasaka et al., 2022; Wang et al., 2023). Consistent with these findings, a previous bovine study reported that a *NANOS3* KO female fetus at 190 days of fetal age displayed a complete absence of germ cells. In alignment with this body of research, the CRISPR/Cas9 *NANOS3* mosaic KO Heifer #854 in this present study was found to be germline ablated as evidenced by the lack of oogenesis observed in her ovarian tissue sections (Figures 11D–J). This observation provides further confirmation that *NANOS3* is necessary for female bovine germline development. Despite the expectation of smaller ovaries based on murine and porcine studies, heifer #854 exhibited contrasting results with abnormal gonad development, characterized by one atrophied ovary and another underdeveloped structure resembling a primitive streak (Figures 11A–C). It should be noted that the ovaries of the *NANOS3* KO female fetus in the previous bovine study, conducted at 190 days of fetal age, were reported to be similar in size to WT ovaries (Ideta et al., 2016). However, it is important to consider the differing developmental timepoints evaluated between these bovine studies. For instance, prior to puberty, *NANOS3* KO pig ovaries were observed to have no discernible differences in appearance or size compared to their age-matched, control, WT counterparts (Kogasaka et al., 2022; Wang et al., 2023). Yet, at the post-puberty stage (6 months old), the *NANOS3* KO pig ovaries were noticeably smaller than those of WT pigs (Kogasaka et al., 2022; Wang et al., 2023), which aligns with the findings observed in our current study in post-puberty bovines.

A potential explanation for the differing outcomes between to *NANOS3* KO bulls and heifers, is the discrepancy in the timing of male and female germline development. During mammalian fetal development, female germ cells start meiosis I and primordial follicles develop with primary oocytes arrested in prophase I at birth. Whereas male germ cells do not start meiosis until after birth, during puberty. Due to the advanced progression of the mammalian female germline during fetal development, it is reasonable to expect that the absence of germ cells in the *NANOS3* KO heifer would lead to more pronounced changes in the reproductive phenotype compared to the *NANOS3* KO bull. Importantly, Ideta et al. (2016) provided evidence that this KO phenotype can be rescued when they successfully generated exogenous primary oocytes and primordial follicles in sterile *NANOS3* KO fetal (141 days) bovine ovaries *via* blastocyst complementation involving the microinjection of WT donor blastomeres into a *NANOS3* KO host.

Finally, meat composition from the two 15 months *NANOS3* KO animals (heifer #854 and bull #838) was analyzed. This was done in part due to the regulatory requirements around food use of gene editing (GnEd) animals, which require a demonstration of low food safety risk to allow products to enter the food supply under enforcement discretion or investigational food use authorization. The results showed the meat composition from these cattle by proximate analysis and mineral content was within the normal variation seen in international databases (Trott et al., 2022). This is not surprising as a *NANOS3* KO would not be expected to alter meat composition.

Taken all together, these findings suggest that *NANOS3* KO cattle could serve as hosts for germline complementation strategies. Germline complementation is the concept of using donor cells from one genetic background to complement or replace the germline of an otherwise sterile host of a different genetic background. By introducing germ cells from high genetic merit donors into the gonads of infertile hosts, it becomes possible to expand the availability of gametes from genetically desirable dams and sires, which could be of benefit to livestock breeding programs (Gottardo et al., 2019). This approach, also known as surrogate sires, or absolute transmitters, offers several advantages. Firstly, in extensively managed systems such as beef cattle, surrogate sires could enable the transmission of desirable donor genetics through natural mating, facilitating the rapid dissemination of superior genetic traits and potentially decreasing the lag between the genetic merit of the elite seedstock sector and that of the commercial sector. Secondly, donor cells could additionally be GnEd to allow for the targeted introgression of beneficial traits (Gottardo et al., 2019; Bishop and Van Eenennaam, 2020; McLean et al., 2020; Mueller and Van Eenennaam, 2022; Ledesma et al., 2023; Oback and Cossey, 2023). Germline complementation can be achieved through two strategies: testis complementation and embryo complementation, and the timing of germ cell loss in the host and donor cell source determines which complementation strategy can be used.

Transplantation of SSC into the sterile testes of males has been achieved in *Dazl* KO mice and rats (Speed et al., 2003; Richardson et al., 2009), *Etv5* KO mice (Zhang et al., 2021), *NANOS2* KO mice, boars, bucks, and bulls (Ciccarelli et al., 2020; Latham et al., 2023), *NANOS3* KO boars (Wang et al., 2023), and *Tscd22d3* KO mice (Zhou et al., 2019). Germline complementation *via* embryo complementation has been achieved in *NANOS3* KO male mice (Miura et al., 2021), a *NANOS3* KO heifer of fetal-age (Ideta et al., 2016), *Prdm14* KO rodents (Kobayashi et al., 2021), and *Tscd22d3* KO mice (Koentgen et al., 2016).

A comparison of germline complementation studies indicates that embryo complementation produced a higher proportion of fertile animals transmitting the donor-derived genotype compared to testis complementation (Oback and Cossey, 2023). Additionally, embryo-mediated, germline complemented murine sires do not differ from regular sires in terms of onset of sexual maturity and fertility (Miura et al., 2021). Embryo complementation presents a unique challenge known as sex chimerism, where female donors may unintentionally be combined with male hosts or *vice versa*, potentially resulting in a hermaphrodite phenotype. However, this concern can be effectively addressed by exclusively combining host and donors of the same sex, either through using sexed sperm or by employing PCR-based sex identification techniques for precise selection of complementation partners.

We propose that *NANOS3* is an excellent target for generating germline-ablated hosts in cattle for germline complementation, for two primary reasons. Firstly, *NANOS3* is one of the earliest genes expressed specifically in PGCs. Therefore, its disruption would eliminate PGCs at an earlier stage compared to other targets like *NANOS2* or *DAZL*, which enables the use of embryo complementation. Secondly, here we have demonstrated that *NANOS3* plays an essential role in both male and female germ cell development, making *NANOS3* KO cattle viable hosts for producing donor-derived germ cells in both sexes. This presents an opportunity to expand the availability of gametes from both genetically desirable sires and dams, thus reducing the genetic lag that exists between the seedstock and commercial sectors of the beef industry. Importantly, if the donor line is unedited, the offspring of GnEd surrogate hosts would not carry any edits and would be classified as null-segregants from a regulatory perspective. However, editing of the donor line might be advantageous in some situations, especially in the generation of homozygous GnEd offspring of both sexes which would be of particular importance for the introduction of recessive GnEd traits into a breeding program.

### 4.1 Limitations of this study and future directions

Sample evaluation in this study began at 41 days, which falls after the expected completion of PGC migration, future studies should delve into the mechanism and timing of fetal germ cell loss by evaluating earlier stages of fetal development. Additionally, employing techniques such as tunnel assays for apoptosis would provide valuable insights into the fate of PGCs, shedding light on the number of PGCs that reach the genital ridge before undergoing cell death. This study did not analyze hormone levels during fetal development of *NANOS3* KO bovines, which limits our understanding of the potential impacts of the absence of *NANOS3* on gonadal development. Investigating hormone profiles during fetal development would provide a more comprehensive picture of the effects of *NANOS3* disruption, particularly in female animals where germline development progresses further during the fetal stage compared to males. To further elucidate the role of *NANOS3* in cattle, future studies could focus on targeting specific mutations in the N-terminal and zinc finger domains of the protein. This approach would help determine if either or both of these domains have essential roles, similar to what has been observed in mice, and provide insights into the functional importance of specific regions of the *NANOS3* protein.

Ultimately, to fully understand the potential applications and suitability of *NANOS3* KO cattle as surrogate hosts, germline complementation experiments will need to be conducted in both sexes. Comparisons can be made between testis complementation in juvenile bulls and embryo complementation to assess the success of restoring gametogenesis in sterile host animals. Future studies will be required to determine if *NANOS3* KO heifers complemented with germline-competent donor cells can produce follicles and functional oocytes. Such studies would provide valuable information on the functionality and fertility potential of *NANOS3* KO animals and their suitability as hosts for germline complementation and transmission.

## 5 Conclusion

This study demonstrates that the absence of *NANOS3* in cattle leads to the specific deficiency of both male and female germ cells. The elimination of germ cells in *NANOS3* KO testes as early as 41 days suggests a conserved role of *NANOS3* in promoting bovine PGC survival, similar to its function in mice. Importantly, we demonstrate that despite the lack of germ cells, seminiferous tubule development was not impaired in *NANOS3* KO bovine testes during fetal, perinatal, and adult stages. Furthermore, the live *NANOS3* KO bull exhibited normal reproductive development and pre-pubertal hormone levels despite the absence of germ cells. These findings highlight the potential of *NANOS3* KO bulls as hosts in germline complementation strategies. In addition, our successful production of a live, germline-ablated, *NANOS3* KO, heifer combined with the previous report of an embryo complemented *NANOS3* KO bovine female fetus (Ideta et al., 2016), support the potential use of *NANOS3* KO heifers to also serve as hosts in germline complementation strategies. Therefore, *NANOS3* KO cattle could be hosts for donor-derived exogenous germ cell production in both sexes, which could provide an opportunity to expand the availability of gametes from both genetically desirable sires and dams, potentially enabling the efficient generation of absolute transmitters of homozygous GnEd gametes of both sexes. Overall, our findings contribute to the understanding of *NANOS3* function in cattle and have valuable implications for the development of novel breeding technologies using germline complementation.

## Data Availability

The data presented in the study are deposited in the NCBI Sequence Read Archive (SRA) repository, accession numbers SAMN37734900–SAMN37734907, and in the NCBI Gene Expression Omnibus (GEO) repository, accession number GSE244321.
