# Supplementary material for: Germline ablation achieved via CRISPR/Cas9 targeting of NANOS3 in bovine zygotes
Source: Front Genome Ed. 2023 Nov 27;5:1321243. doi: 10.3389/fgeed.2023.1321243 (PMC10711618; doi:10.3389/fgeed.2023.1321243)
Supplement: Supplementary file 1 [file Table1.DOCX]

Supplementary Material

**Supplementary Table 1.** List of single guide RNA (sgRNA) sequences tested for targeting bovine *NANOS3*

| Guide name | Location  (exon 1) | Sequence (20 nt sgRNA + PAM) |
| --- | --- | --- |
| 1 (sgRNA1) | 5’ | GTGGACAGACTACTTGGGTTTGG |
| 2 (sgRNA2) | 5’ | CTACTTGGGTTTGGCACGCCTGG |
| 3 (sgRNA3) | 5’ | GTTCGGGCACTGCTTCTGGCTGG |
| 4 (sgRNA4) | 5’ | AGAAGCAGTGCCCGAACCGGGGG |
| 5 (sgRNA5) | Center | CGCTTCATCCTTGAGCACGTGGG |
| 6 (sgRNA6) | 3’ | TGGTCCGCTCGGACAAGGCGAGG |
| 7 (sgRNA7) | 3’ | CTCGGACAAGGCGAGGACGCAGG |

**Supplementary Table 2.** List of PCR primer sequences. Forward (fwd); Reverse (rev)

| **Name** | **Target** | **Type** | **Expected amplicon (bp)** | **Sequence** | **Notes** |
| --- | --- | --- | --- | --- | --- |
| **NANOS3_F1** | NANOS3, exon 1 | Fwd | 770 | GAACTGACAGCCCAGACTCC | 1st round of nested PCR |
| **NANOS3_R1** | NANOS3, exon 1 | Rev |  | GCTTACCCACTAGGGCAACA |  |
| **NANOS3_F2** | NANOS3, exon 1 | Fwd | 610 | GCGTTTCTCCTGTCTTCTGC | 2nd round of nested PCR |
| **NANOS3_R2** | NANOS3, exon 1 | Rev |  | AACCCTCTGAAGTGGGTCAG |  |
| **NANOS3_6kb_2F** | NANOS3, long-range | Fwd | 6,274 | CCTCAACTGACGGGGAAGTC |  |
| **NANOS3_6kb_2R** | NANOS3, long-range | Rev |  | TTGTTGTCGGTGGGTTGTGA |  |
| **DDX3_F** | DDX3 | Fwd | X = 208; Y = 184 | AGGAAGCCAGGAAAGTAA |  |
| **DDX3_R** | DDX3 | Rev |  | CATCCACGTTCTAAGTCTC |  |
